# Genome-Wide Identification and Expression Analysis of bZIP Family Genes in *Stevia rebaudiana*

**DOI:** 10.3390/genes14101918

**Published:** 2023-10-09

**Authors:** Mengyang Wu, Jinsong Chen, Weilin Tang, Yijie Jiang, Zhaoyong Hu, Dongbei Xu, Kai Hou, Yinyin Chen, Wei Wu

**Affiliations:** Agronomy College, Sichuan Agricultural University, Chengdu 611130, China; wmy330yx@163.com (M.W.); chjs1993@163.com (J.C.); weilintang2021@163.com (W.T.); jyj177ky@163.com (Y.J.); yonghz2023@126.com (Z.H.); xudongbei2006@126.com (D.X.); hking@sicau.edu.cn (K.H.); 14227@sicau.edu.cn (Y.C.)

**Keywords:** *S. rebaudiana*, basic leucine zipper, expression analysis, light, phytohormone, abiotic stress

## Abstract

The basic (region) leucine zippers (bZIPs) are evolutionarily conserved transcription factors widely distributed in eukaryotic organisms. In plants, they are not only involved in growth and development, defense and stress responses and regulation of physiological processes but also play a pivotal role in regulating secondary metabolism. To explore the function related to the bZIP gene family in *Stevia rebaudiana* Bertoni, we identified 105 *SrbZIP* genes at the genome-wide level and classified them into 12 subfamilies using bioinformation methods. Three main classes of *cis*-acting elements were found in the *SrbZIP* promoter regions, including development-related elements, defense and stress-responsive elements and phytohormone-responsive elements. Through protein–protein interaction network of 105 SrbZIP proteins, SrbZIP proteins were mainly classified into four major categories: ABF2/ABF4/ABI5 (SrbZIP51/SrbZIP38/SrbZIP7), involved in phytohormone signaling, GBF1/GBF3/GBF4 (SrbZIP29/SrbZIP63/SrbZIP60) involved in environmental signaling, AREB3 (SrbZIP88), PAN (SrbZIP12), TGA1 (SrbZIP69), TGA4 (SrbZIP82), TGA7 (SrbZIP31), TGA9 (SrbZIP95), TGA10 (SrbZIP79) and HY5 (SrbZIP96) involved in cryptochrome signaling, and FD (SrbZIP72) promoted flowering. The transcriptomic data showed that *SrbZIP* genes were differentially expressed in six *S. rebaudiana* cultivars (‘023’, ‘110’, ‘B1188’, ‘11-14’, ‘GP’ and ‘GX’). Moreover, the expression levels of selected 15 *SrbZIP* genes in response to light, abiotic stress (low temperature, salt and drought), phytohormones (methyl jasmonate, gibberellic acid and salicylic acid) treatment and in different tissues were analyzed utilizing qRT-PCR. Some *SrbZIP* genes were further identified to be highly induced by factors affecting glycoside synthesis. Among them, three *SrbZIP* genes (*SrbZIP54*, *SrbZIP63* and *SrbZIP32*) were predicted to be related to stress-responsive terpenoid synthesis in *S. rebaudiana*. The protein–protein interaction network expanded the potential functions of *SrbZIP* genes. This study firstly provided the comprehensive genome-wide report of the *SrbZIP* gene family, laying a foundation for further research on the evolution, function and regulatory role of the bZIP gene family in terpenoid synthesis in *S. rebaudiana*.

## 1. Introduction

The basic leucine zipper (bZIP) is one of the most widely distributed and conserved transcription factor families among eukaryotes, playing a significant role in regulating plant growth and development. Extensive research has explored bZIP genes in various species, including *Arabidopsis thaliana* (78) [1], poplar (86) [2], *Isatis indigotica* (65) [3], licorice (*Glycyrrhiza uralensis*) (66) [4] and safflower (*Carthamus tinctorius*) (52) [5].

The bZIP domain consists of a basic amino acid region (N-X7-R/K) and a leucine zipper region that binds to the alkaline region [6]. The leucine (Leu) zipper region is located in the N-terminus and consists of highly conserved heptad repeats of leucine or other large hydrophobic amino acids, enabling the formation of homodimers and heterodimers among bZIP proteins through the leucine zipper. In plants, the bZIP proteins are preferentially combined with ACGT core sequences [1]. Jakoby et al. initially divided the members of the bZIP gene family in *A. thaliana* into 10 subgroups (A, B, C, D, E, F, G, H, I, S) based on their conserved domain [6]. Then, Dröge-Laser et al. updated the classification of the *A. thaliana* bZIP-family. The *AtbZIP* genes were further divided into 13 subgroups, adding three groups (M, K and J) [1].

In plants, members of the bZIP TF family participate in multiple biological processes, including tissue and organ development, responses to abiotic and biotic stresses and secondary metabolism regulation. Co-expression of *AtbZIP10*, *AtbZIP25* and *ABI3* regulates seed specificity [7]. *AtbZIP46* determines the number of floral organs and participates in stem and floral meristem expression [8]. *HY5* and *HYH* mediate light response in *A. thaliana* as main regulators of photomorphogenesis [9]. *AtbZIP17* functions as a transcriptional activator in the response to salt stress [10]. *OsbZIP16* can reduce the sensitivity of overexpressed rice seedlings to abiotic stress during germination [11]. Additionally, many studies have shown that bZIP transcription factors effectively regulate the biosynthesis of plant secondary metabolites, such as terpenoids, alkaloids and flavonoids. *AaHY5* regulates light-induced artemisinin biosynthesis by binding to the G-box (CACGTG) site in the promoter of *AaGSW1* [12]. CRISPR/Cas9-mediated knock-out of one allele of *VvbZIP36* in grapevine promotes anthocyanin accumulation [13]. *MdHY5* promotes anthocyanin accumulation by binding to the G-box-2 site in the promoter of *MdMYB10*, thereby regulating the expression of downstream anthocyanin biosynthesis genes [14].

*S. rebaudiana*, a perennial herb of the Asteraceae family, is renowned for being a valuable source of the tetracyclic diterpenoid derivative, natural sweetener steviol glycosides (SGs), known for their high sweetness and low-calorie content. This plant holds not only high edible value but also significant medicinal importance, with steviol glycosides exhibiting various pharmacological effects such as anticancer, anticardiovascular disease, anti-inflammatory and antimicrobial. Despite its increasing application of SGs, the production of *S. rebaudiana* falls short of meeting the market demand, necessitating urgent efforts to enhance production and ensure product quality by conducting insights into its developmental processes and responses to environmental factors. With the rapid development of DNA sequencing techniques, there have been reports of studies sequencing the stevia genome [15], laying a solid foundation for genome-wide analysis of bZIP genes in *S. rebaudiana*.

The multiple biological functions of bZIP proteins are of great interest in plant science. Although bZIP genes have been characterized in other plants [1,2,3,4,5], genome-wide identification and characterization of bZIP of *S. rebaudiana* is yet to be conducted. So far, little is known at present about the connection between bZIP genes and terpenoid biosynthesis in *S. rebaudiana*. In this study, we identified the *SrbZIP* gene family members utilizing bioinformatics methods at the genome level. Additionally, we performed phylogenetic analysis, examined chromosomal locations and collinearity, analyzed motif compositions of *cis*-acting elements in the promoter, studied gene structures, conducted protein interaction network analysis and then explored the expression of *SrbZIP* genes in six *S. rebaudiana* cultivars. Moreover, we investigated the *SrbZIP* gene’s response to various factors related to terpenoid synthesis, including light, abiotic stress (low temperature, salt and drought), phytohormones (methyl jasmonate, gibberellic acid and salicylic acid), as well as in different tissues, utilizing qRT-PCR. The findings from this study offered valuable insights for further potential regulatory role of bZIP genes in the terpenoid biosynthesis of *S. rebaudiana.*

## 2. Materials and Methods

### 2.1. Plant Materials

Six *S. rebaudiana* cultivars (‘GX’, ‘GP’, ‘B1188’, ‘110’, ‘11-14’ and ‘023’) were harvested from the growth room of Sichuan Agricultural University (Chengdu, China) and propagated utilizing a 1:1 mixture of nutrient soil and vermiculite (temperature: 23 °C; photoperiod: 16 h light/8 h darkness; humidity: 70–75%). The cultivars of ‘11-14’ and ‘B1188’ were obtained from Anhui province and Shandong province, respectively. The remaining cultivars were obtained by the induction through mutagenic methods as follows: ‘023’ and ‘110’ were obtained from induction by ^60^Co γ-ray irradiation of the callus of ‘11-14’ leaf tissue; ‘GX’ was induced from the tissue culture seedling of ‘11-14’ in medium with high concentration of zinc; ‘GP’ was induced from the tissue culture seedling of ‘11-14’ in medium with high concentration of boron. All samples were identified by Professor Wei Wu, who studied *S. rebaudiana* for more than 10 years. The ‘023’ *S. rebaudiana* cultivar contains more types of stevia glycosides, so we chose it as the experimental material. Different *S. rebaudiana* tissue samples (root, stem, leaf and flower) were collected in triplicate to explore the discrepancy of expression patterns of *SrbZIP* genes in various tissues. The samples were snap-frozen in liquid nitrogen and then stored at −80 °C.

Furthermore, we evaluated the expression levels of *SrbZIP* genes under light, abiotic stress (low temperature (4 °C), salt (NaCl) and drought (PEG) and phytohormones treatment (salicylic acid, Methyl jasmonate and gibberellic acid). We selected eighteen seedlings (three biological replicates were used for each treatment, six seedlings were treated in each replicate) for each treatment, these eighteen stevia seedlings were treated by foliar spraying with 200 mM NaCl [16], 5% PEG 4000 [17], 2 mM SA [18], 0.1 mM MeJA [19] and 2 mg·L^−1^ GA [20], and samples (the third leaf tissues) were collected at indicated time points (0, 1, 3, 6, 12, 24 and 48 h) after treatment. For light treatment, eighteen stevia seedlings were grown under normal light illumination (150 µm^−2^s^−1^), while another set of eighteen stevia seedlings under a dark environment, and samples (the third leaf tissues) were collected at indicated time points (0, 1, 3, 6, 9, 12, 24 and 48 h) after treatment (added 9 h time point). For low-temperature treatment, stevia plants were grown at 4 °C, and other growth conditions were the same [21]. Three biological replicates were used for each treatment, and the 0 h treatment was set as the control. The samples were snap-frozen in liquid nitrogen and then stored at −80 °C.

### 2.2. Data Sources

The complete genome sequences for *S. rebaudiana* were retrieved from the NCBI database (http://www.ncbi.nlm.nih.gov/, accessed on 14 February 2023). The RNA transcriptome data were used from our previous study [22]. The AtbZIP protein sequences were obtained from the PlantTFDB database (http://planttfdb.gao-lab.org/, accessed on 1 March 2023).

### 2.3. Identification of S. rebaudiana bZIP Gene Family

First, to classify the *SrbZIP* gene family members, the known *AtbZIP* sequences were used to query the homologous sequences in the *S. rebaudiana* genome by performing BLASTp searches (E-value < 10^−14^). Second, the profile hidden Markov models (HMMs) of the bZIP domain (PF00170, PF07716, PF12498 and PF03131) were downloaded from the Pfam database (http://pfam.xfam.org/, accessed on 2 March 2023). Then, we further identified and screened the conserved domains utilizing the NCBI Conserved Domain Search (CD-Search) Tool (https://www.ncbi.nlm.nih.gov/Structure/cdd/wrpsb.cgi, accessed on 5 March 2023). After removing incomplete and redundant sequences, members of the *SrbZIP* gene family were obtained. The identified amino acid sequence was submitted to the ProtParam tool of the ExPASy website (http://web.expasy.org/protparam/, accessed on 5 March 2023) for predictions of physicochemical properties and to Wolf Psort website (https://www.genscript.com/wolf-psort.html, accessed on 5 March 2023) for protein subcellular localization prediction.

### 2.4. S. rebaudiana bZIP Chromosomal Location and Collinearity Analysis

We mapped the sequence of the *SrbZIP* genes to individual chromosomes by analyzing *S. rebaudiana* genome annotation information and using TBtools (v1.098) (https://github.com/CJ-Chen/TBtools-Manual, accessed on 8 March 2023) [23,24]. We employed MCScanX (v1.098) software to investigate gene duplication events [25]. Two genes located on the same chromosomal fragment were separated by five genes or less, and the distance between them was less than 100 kb; these were considered tandem duplicated genes [26]. The collinearity relationships between the *SrbZIP* genes and related genes were visually analyzed utilizing TBtools software (v.1.098).

### 2.5. Phylogenetic Analysis of SrbZIP Genes

To explore the phylogenetic relationships between the bZIP proteins of *S. rebaudiana* and *A. thaliana*, we used the neighbor-joining (NJ) method constructed in MEGA11 software (v11.0.13) [27]. We constructed a multiple alignment of the amino acid sequences by using ClustalW. The phylogenetic analysis included 1000 bootstrap replicates and was visualized using the Interactive Tree of Life (iTol) server (https://itol.embl. de/, accessed on 20 March 2023).

### 2.6. Gene Structure, Conserved Motif and Cis-Acting Element Analysis

The conserved motifs of bZIP proteins were analyzed utilizing the MEME Suite tools (https://meme-suite.org/meme/tools/meme, accessed on 6 May 2023) [28]. The upstream 2.0 kb sequence of each bZIP gene was defined as the promoter region, and the promoter sequence was retrieved and submitted to the PlantCARE website (http://bioinformatics.psb.ugent.be/webtools//plantcare/html/, accessed on 6 May 2023). Visualization of the conserved domains, exons, introns, motifs and *cis*-elements in the bZIP genes was performed utilizing the TBtools software package (v1.098) [24].

### 2.7. Protein–Protein Interaction Analysis Network of SrbZIPs

We retrieved SrbZIP proteins with the highest homology to AtbZIP proteins using OrthoVenn software (v3.0) with the following conditions: E-value 1 × 10^−2^ and inflation value 1.5 (https://orthovenn3.bioinfotoolkits.net, accessed on 7 May 2023). We carried out an interaction prediction network map utilizing the STRING database (https://string-db.org, accessed on 7 May 2023) [29].

### 2.8. S. rebaudiana bZIP Expression Pattern Analysis

Reads from eighteen samples of six different genotypes (‘GX’, ‘GP’, ‘B1188’, ‘110’, ‘11-14’ and ‘023’) were produced (six different genotypes of leaf tissues with three technical repetitions). The expression analysis from the reads of different samples was carried out with bowtie2 software (v3.3.3.1) by calculating the fragments per kilobase million (FPKM) value of the expression, and the (FPKM) value of each *SrbZIP* gene was retrieved from the RNA-seq data [22]. TBtools software (v1.098) was used to analyze the expression pattern by constructing heatmaps of *SrbZIP* gene expression.

The total RNA was extracted using an RNA extraction kit (TRIzol, Vazyme) and the quality and concentration of the RNA were determined utilizing the Termo NanoDrop 2000, then reverse-transcribed to cDNA using a HiScript II 1st strand cDNA synthesis kit (Vazyme). The candidate *SrbZIP* genes were selected for qRT-PCR validation analysis, which was performed utilizing BlasTaq™ 2X qPCR MasterMIX (abm), and the stevia *β-actin* [AF548026] gene was used as an internal reference gene. The 20 μL reaction mixture contained 1 μL cDNA and 0.5 μL each primer and 10 μL master mix (BlasTaq™ 2X qPCR Mastermix), water blank control. The cycling conditions were as follows: enzyme activation at 95 °C for 3 min; then 40 cycles at 95 °C for 15 s (denaturation), 60 °C for 60 s (extension), followed by a melting curve analysis. Relative transcript abundances were calculated via the 2^−ΔΔCt^ method [30]. The primers are listed in Table S1. All data were normalized based on setting up the relative expression level. The expression level of 0-point treatments for phytohormones and abiotic stress was set as 1.

### 2.9. Statistical Analysis

Visualization and statistical analysis of data were performed by GraphPad (v8.0.2). We conducted a mixed effects model (treatment effect, time effect and treatment/time interaction effect). Correct for multiple comparisons using Bonferroni’s multiple comparisons test. Significant differences between the treatment group and control group are denoted by asterisk(s) (** *p* < 0.01, * *p* < 0.05).

## 3. Results

### 3.1. Identification and Characterization of the SrbZIPs

A comprehensive search and structural domain determination led to the identification of 105 bZIP family members in *S. rebaudiana*, named *SrbZIP1-SrbZIP105*. Analysis of the amino acid physicochemical properties showed that the amino acid length readings varied from 96 aa (SrbZIP13) to 704 aa (SrbZIP17), and the corresponding molecular weight size ranged from 10.88 kDa (SrbZIP13) to 77.31 kDa (SrbZIP17). The theoretical isoelectric point (pI) of SrbZIP proteins ranged from 4.64 (SrbZIP21) to 10.08 (SrbZIP101). The hydrophilic nature of the 105 sequences was indicated by their GRAVY values, with a maximum value of −0.24 (SrbZIP3) and a minimum value of −1.425 (SrbZIP27). The aliphatic index of SrbZIPs ranged from 37.60 (SrbZIP38) to 104.04 (SrbZIP88). Instability index analysis revealed that, except for five proteins (SrbZIP1, SrbZIP23, SrbZIP51, SrbZIP62, SrbZIP95), all SrbZIP proteins were predicted to be unstable, as they exceeded 40 [31]. The majority of SrbZIP proteins were predicted to be located in the nucleus, while three SrbZIP proteins (SrbZIP4, SrbZIP25, SrbZIP27) were predicted to be localized in cytoplasm, two SrbZIP proteins (SrbZIP12, SrbZIP17) in endoplasmic reticulum, and one SrbZIP protein (SrbZIP2) in chlorosomes through subcellular localization prediction. The proteins that were neutral or alkaline accounted for 97.14% (102/105) of the total number (Table S2).

### 3.2. Classification of SrbZIP Genes Based on Phylogram

**Figure 1:**
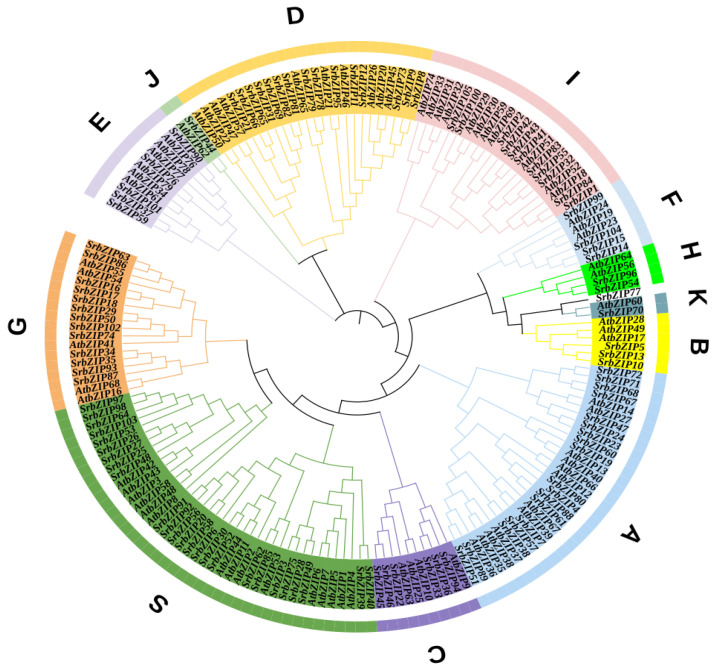
Phylogenetic tree of the bZIPs from *S. rebaudiana* and *A. thaliana*. The capital letters denote different subgroups.

To investigate the evolutionary connections and categorization of the bZIP family, we constructed a neighbor-joining (NJ) phylogenetic tree utilizing the MEGA11 software (v11.0) with 1000 bootstrap replicates. The phylogenetic tree comprised 105 *SrbZIPs* and 73 *AtbZIPs*. *S. rebaudiana* bZIP genes were divided into 12 subgroups (*SrbZIP*-A, *SrbZIP*-B, *SrbZIP*-C, *SrbZIP*-D, *SrbZIP*-E, *SrbZIP*-F, *SrbZIP*-G, *SrbZIP*-H, *SrbZIP*-I, *SrbZIP*-J, *SrbZIP*-K and *SrbZIP*-S) referring to the principles of classification in *A. thaliana* [1], (Figure 1). However, *SrbZIP77* could not be aggregated into any subfamily, and we speculate that the structure of the *SrbZIP77* has been greatly different in evolution or differed from the evolutionary direction of *AtbZIPs*. The *SrbZIP* genes classified in subgroup S contained the largest number of members, including 25 in *S. rebaudiana* and 15 in *A. thaliana*, while subgroup J and subgroup K contained the fewest members, both including only one in *S. rebaudiana*.

### 3.3. Chromosomal Location and Collinearity Analysis of SrbZIP Members

**Figure 2:**
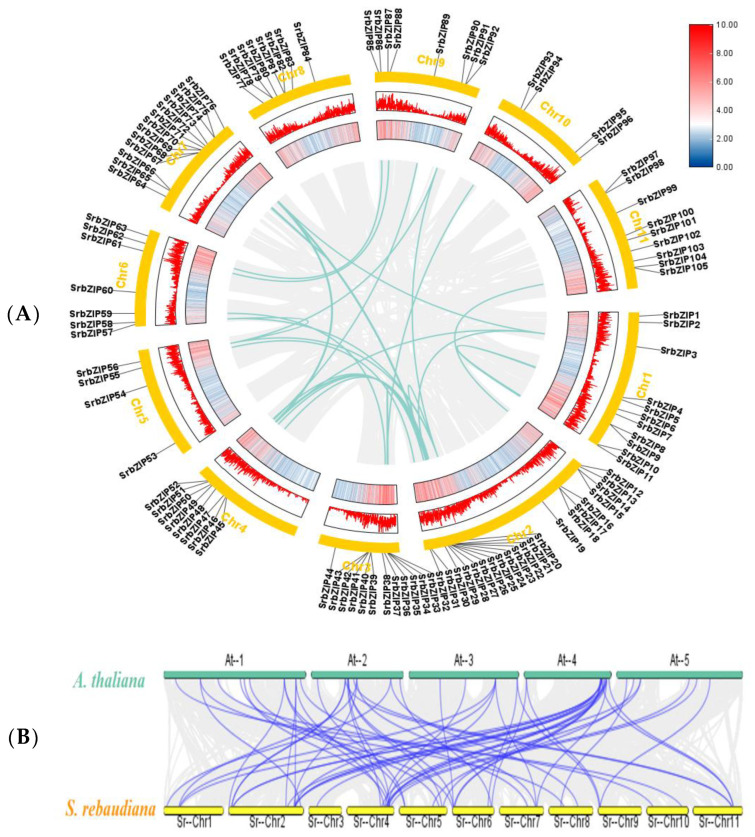
(**A**) Chromosomal distribution and gene duplication of *SrbZIP* genes. The 11 chromosomes of *S. rebaudiana* are represented in yellow circles, blue lines connect homologous genes and gray lines represent collinear pairs in the *S. rebaudiana* genome. (**B**) Syntentic analysis of bZIP genes between *S. rebaudiana* and *A. thaliana*. The blue lines represent collinear bZIP gene pairs, while gray lines in the background represent the collinear blocks.

The distribution of the 105 *SrbZIP* genes is across 11 chromosomes (Chr1-Chr11) in *S. rebaudiana* (Figure 2A). Notably, chromosome 2 carried the highest number of *SrbZIP* genes (21), whereas chromosomes 5 and 10 had the fewest *SrbZIP* genes (4) (Figure 2A). Surprisingly, the number of *SrbZIP* genes on each chromosome was independent of the chromosome size.

Gene duplication events play important roles in the evolution and expansion of gene families and generally occur through two major mechanisms: segmental and tandem duplications [32]. To analyze the expansion pattern of *SrbZIP* genes, we analyzed their gene duplication events utilizing MCScanX. Twenty-seven pairs of segmental duplication events occurred on eleven chromosomes (Figure 2A; Table S3). Additionally, *SrbZIP67* and *SrbZIP68* genes experienced tandem duplication events (Table S4) [26]. The most common segmental duplication event in plants leads to the expansion of family members on different chromosomes [26]. Our analysis showed that most of the *SrbZIP* genes were generated by segmental duplication, which was the major driving force for *SrbZIP* gene expansion (Table S4).

In this study, interspecies collinearity analysis between *SrbZIPs* and *AtbZIPs* was constructed using Circle Gene View and Dual Systeny Plot for MCScanX. The results showed a conservative collinearity relationship with 62 orthologous pairs found between *S. rebaudiana* and *A. thaliana*. The segmental duplications and the collinearity between members of two gene families may have played vital roles in the evolution of the bZIP gene family (Figure 2B; Table S5).

### 3.4. Gene Structure and Conserved Motif Analysis of SrbZIPs

**Figure 3:**
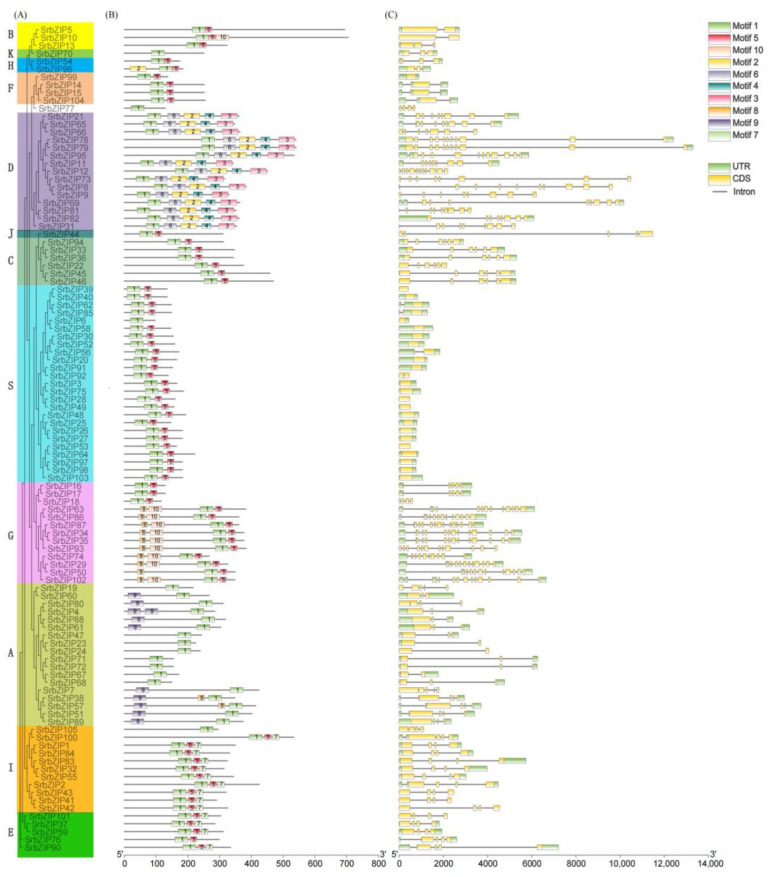
Phylogenetic tree, motif composition and gene structure for *SrbZIP* TFs. (**A**) Phylogenetic tree of *SrbZIPs*. (**B**) Motif distribution. (**C**) The exon-intron distribution in *SrbZIP* genes. Yellow boxes indicate untranslated region (UTR), green boxes represent coding sequence (CDS) and gray lines indicate introns.

To investigate the gene structure and the motif composition of *SrbZIPs*, the phylogenetic tree, motif and gene structure were built using TBtools (v1.098) (Figure 3). The analysis identified ten conserved motifs (Figure S1), with characteristic motif 1 regions found in all bZIP family members of *S. rebaudiana* (Figure 3B), indicating high conservation during the evolution of SrbZIP proteins. Motif 5 regions were detected in most bZIP subfamilies, except subfamily A and D. Gene structures within the same bZIP subfamily were mostly identical, although some exceptions were observed, for example, motif 9 regions were absent in seven bZIP members (*SrbZIP47*, *SrbZIP23*, *SrbZIP24*, *SrbZIP71*, *SrbZIP72*, *SrbZIP67*, *SrbZIP68*) of subgroup A compared to others. The C-terminal regions containing more motifs were crucial for bZIP dimerization and DNA binding (Figure 3B). All SrbZIP proteins were found to have the bZIP superfamily domain (including BRLZ, MFMR and DOG1 domains), and six SrbZIP proteins contained the bZIP-HY5-like domain (SrbZIP5, SrbZIP10, SrbZIP13, SrbZIP70, SrbZIP54, SrbZIP96) (Figure S2). Moreover, we investigated the exon-intron distribution patterns in *SrbZIP* genes (Figure 3C). Around 69.52% of the *SrbZIP* gene members contained 1–5 introns. The members of subgroup G, except *SrbZIP16*, *SrbZIP17* and *SrbZIP18*, contained more than ten introns. A maximum of 13 introns were detected in *SrbZIP87*. Moreover, members of the same *SrbZIP* subgroup displayed similar intron/exon structures. For instance, most *SrbZIP-*S genes had no intron, while two members of subgroup H both contained two introns.

### 3.5. Cis-Elements Analysis in SrbZIPs Promoter Regions

**Figure 4:**
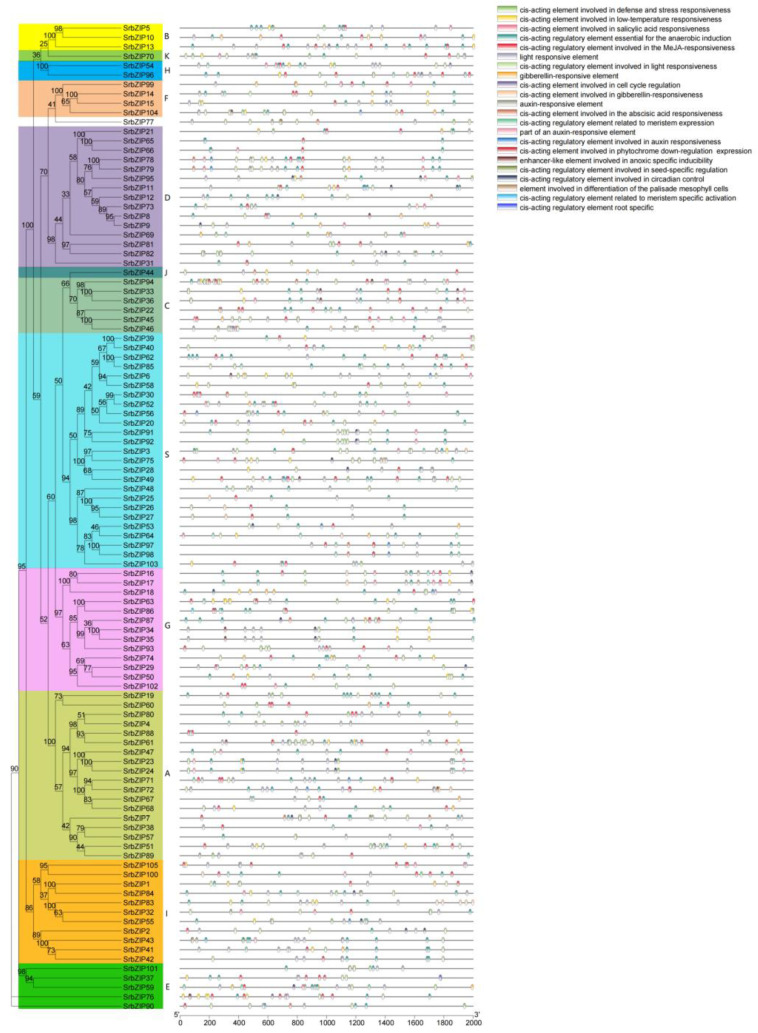
*Cis*-acting elements for *SrbZIP* promoters in *S. rebaudiana*.

The promoter regions located within 2.0 kb upstream of *SrbZIPs* were submitted to the PlantCARE database for predicting *cis*-elements to understand the regulatory mechanisms underlying the response of *SrbZIPs* to abiotic or biotic stress. The *cis*-elements in the *SrbZIP* promoters fell into three main categories: development-related elements, defense and stress-responsive elements and phytohormone-responsive elements (Table S6). Most environmental stress-related elements were light-responsive elements, drought induction (MBS), low-temperature-responsive elements (LTR) and antioxidant response elements (ARE) (Figure 4). In addition, there were many phytohormone-responsive elements such as abscisic acid responsiveness (ABRE), MeJA-responsive elements (TGACG-motif), salicylic acid-responsive elements (TCA-element) were identified to exist in *SrbZIP* promoters. The members of subgroup G contained abundant light-responsive elements, and the *SrbZIP93* promoter region contained 20 light-responsive elements. Subgroup A members, particularly *SrbZIP61*, exhibited an elevated number of abscisic acid-responsive elements (ABREs), with 13 ABREs identified.

### 3.6. Protein Interaction Analysis Network of SrbZIP Proteins

**Figure 5:**
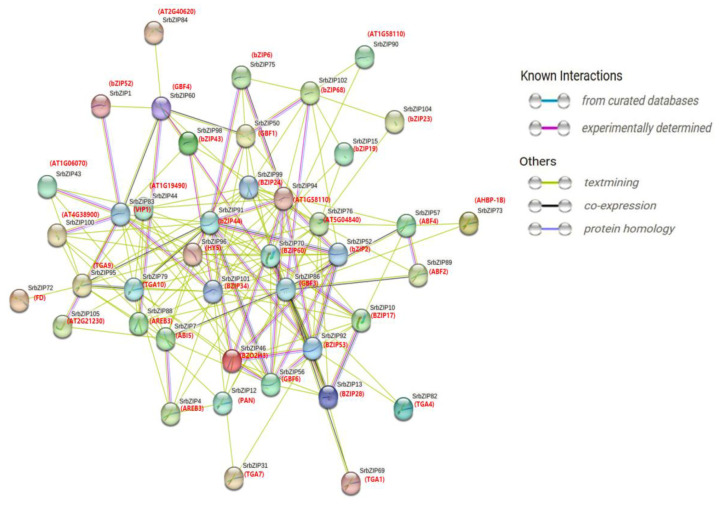
Protein–protein interaction prediction network of SrbZIP proteins. The network nodes represent proteins, and the line colors indicate the types of interaction evidence.

To explore the interactions among SrbZIP proteins, we constructed a protein–protein interaction network (*p*-value: <1.0 × 10^−16^) relationship map of 105 SrbZIP proteins. The SrbZIPs were a complex family with 43 nodes and 172 edges. In the map of the SrbZIP proteins interaction network, the results showed that 13 members (SrbZIP60, SrbZIP95, SrbZIP89, SrbZIP12, SrbZIP31, SrbZIP7, SrbZIP4, SrbZIP57, SrbZIP88, SrbZIP79, SrbZIP73, SrbZIP82 and SrbZIP69) were predicted to be involved in plant hormone signal transduction (ath04075), of which 3 members (SrbZIP7, SrbZIP57 and SrbZIP89) related to sugar and hormone signaling (WP3661). In addition, 4 SrbZIP proteins (SrbZIP83, SrbZIP88, SrbZIP7 and SrbZIP102) of the network of SrbZIP protein were predicted to be associated with seed development (WP2279). Based on their functions, SrbZIP proteins were categorized into four main groups: ABF2/ABF4/ABI5 (SrbZIP51/SrbZIP38/SrbZIP7), involved in phytohormone signaling: GBF1/GBF3/GBF4 (SrbZIP29/SrbZIP63/SrbZIP60) involved in environmental signaling, AREB3 (SrbZIP88), PAN (SrbZIP12), TGA1 (SrbZIP69), TGA4 (SrbZIP82), TGA7 (SrbZIP31), TGA9 (SrbZIP95), TGA10 (SrbZIP79) and HY5 (SrbZIP96) involved in cryptochrome signaling, and FD (SrbZIP23, SrbZIP24, SrbZIP47, SrbZIP67, SrbZIP68, SrbZIP71, SrbZIP72) promoting flowering (Figure 5; Table S7).

### 3.7. Expression Pattern and qRT-PCR Validation of SrbZIP Genes

**Figure 6:**
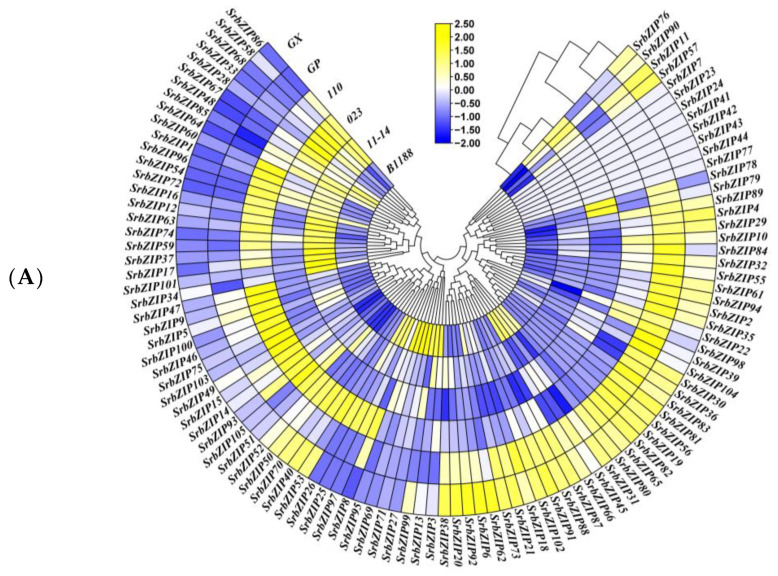

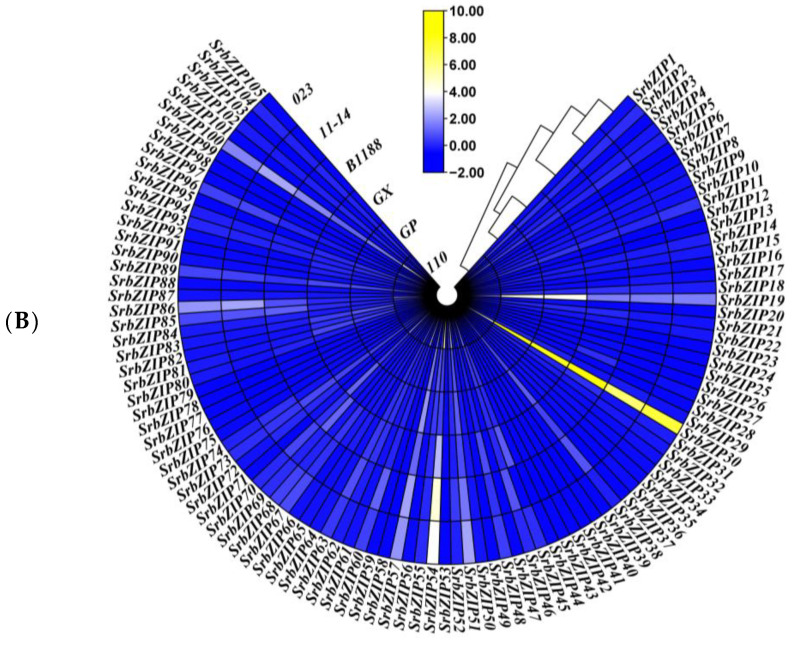
(**A**) Heatmap depicting the expression patterns of *SrbZIP* genes in *S. rebaudiana* leaves from six cultivars. The FPKM values were subjected to rowscaling transformation. (**B**) Cluster columns (six varieties). Yellow indicates higher expression, and blue represents lower expression.

The fragments per kilobase million (FPKM) values of the *SrbZIP* genes were retrieved from the transcriptome data described in our previous study [22] to investigate the expression pattern of *SrbZIP* genes in leaves from different cultivars, and the heatmaps were generated based on these values (Figure 6; Table S8).

*SrbZIP30* and *SrbZIP54* showed higher expression in 6 varieties of *S. rebaudiana* leaves, while *SrbZIP30* in leaves had the highest expression. *SrbZIP30*, a member of subgroup S, was functionally interrelated in plant starvation signaling [1]. The high expression of *SrbZIP30* may provide the most basic conditions in *S. rebaudiana* growth. Likewise, *SrbZIP54* was classified in subgroup H, which was composed of only two members, elongated hypocotyl 5 (*HY5*) and *HY5* homolog (*HYH*). Meanwhile, *HY5* has conclusively proved to be one of the most predominant transcriptional regulators involved in promoting photomorphogenesis, pigment accumulation downstream of phytochromes and chloroplast development [33,34]. Performing as a master regulator in coordinating light, developmental and environmental signaling results in its highly expressed.

In order to further reveal the biological function of *SrbZIP* genes, the expression patterns of *SrbZIP* genes in different tissues and conditions were analyzed using qRT-PCR. We preliminary selected 10 *SrbZIP* genes from different bZIP subfamilies that expressed higher of ‘023’ *S. rebaudiana* cultivar. *SrbZIP30* (from the S subfamily), *SrbZIP54* (from the H subfamily), *SrbZIP100* (from the I subfamily), *SrbZIP60* (from the A subfamily), *SrbZIP63* (from the G subfamily), *SrbZIP70* (from the K subfamily), *SrbZIP32* (from the I subfamily), *SrbZIP9* (from the D subfamily), *SrbZIP21* (from the D subfamily) and *SrbZIP10* (from the B subfamily) have higher expression in 8 bZIP subfamilies of *S. rebaudiana* leaves, in descending order (Figure 6). Comparative DEG analysis revealed that five bZIP genes (*p* < 0.01) significantly differed between the ‘023’ cultivar bZIP genes and the other five cultivars. Among them, *SrbZIP45* (log2(FC) = 4.14 *p* < 0.01, from the C subfamily), *SrbZIP93* (log2(FC) = 3.016 *p* < 0.001, from the G subfamily), *SrbZIP102* (log2(FC) = 2.13 *p* < 0.001, from the G subfamily), *SrbZIP19* (log2(FC) = 5.3 *p* < 0.01, from the A subfamily) and *SrbZIP104* (log2(FC) = 5.14 *p* < 0.001, from the F subfamily) were up-regulated genes with a higher FC cutoff (log2(FC) > 2). We selected 15 up-regulated and highly expressed *SrbZIP* genes from 10 subfamilies, which were gathered to analyze the expression patterns via qRT-PCR in different tissues (root, stem, leaf and flower).

**Figure 7:**
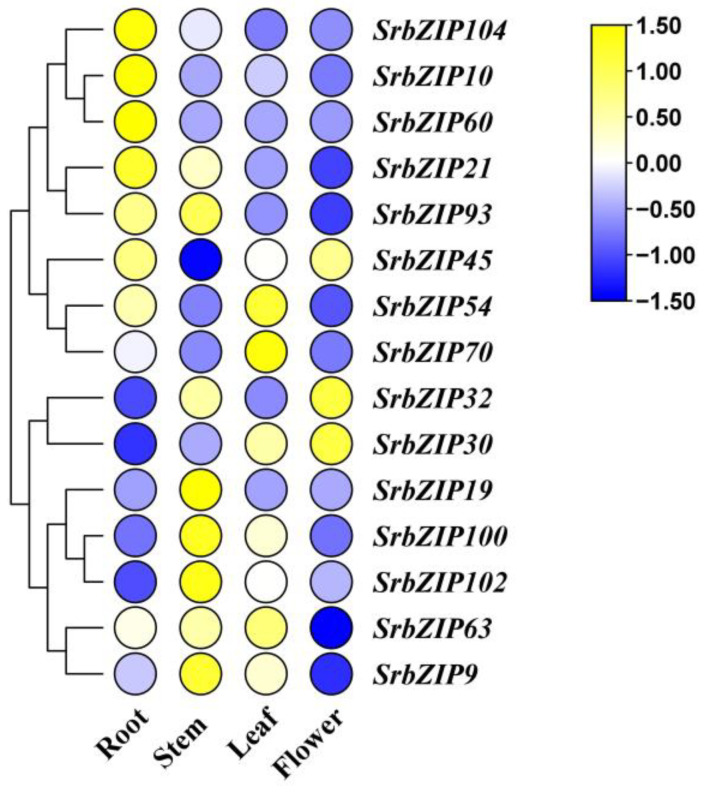
Expression profiles of *SrbZIP* genes in various tissues assessed utilizing qRT-PCR. The data were normalized relative to mean expression value of each gene across all tissues, and the expression levels range from low expression (blue) to high expression (yellow).

The expression levels of most of the 15 *SrbZIP* genes varied considerably among different tissues (Figure 7). Among the examined 15 *SrbZIP* genes, 3 *SrbZIP* genes (*SrbZIP63*, *SrbZIP54*, *SrbZIP70*) showed higher expression levels in leaves, 3 *SrbZIP* genes (*SrbZIP60*, *SrbZIP104*, *SrbZIP10*) presented high expression in roots, 5 *SrbZIP* genes (*SrbZIP93*, *SrbZIP100*, *SrbZIP102*, *SrbZIP9*, *SrbZIP19*) presented high expression in stems and *SrbZIP30* presented high expression in flowers. These findings indicate that these screened *SrbZIP* genes have tissue-specific expression potentials in *S. rebaudiana*.

### 3.8. Expression Pattern of the SrbZIP Genes in Response to Phytohormones and Abiotic Stresses

**Figure 8:**
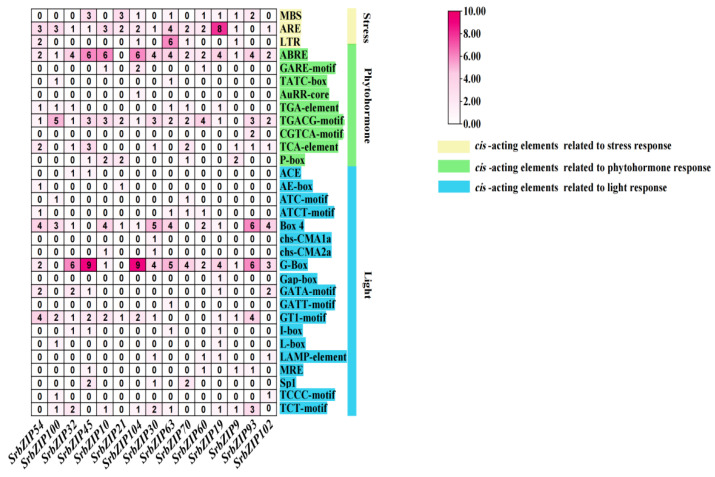
Distribution of *cis*-elements in the upstream regions of promoters of the 15 *SrbZIP* genes in *S. rebaudiana.* The *cis*-acting elements filled with yellow are related to stress response, the *cis*-elements filled with green are related to phytohormone response and the *cis*-elements filled with blue are related to light response.

Based on the factors that affect the synthesis of stevia glycosides [35], we found that stevia glycosides varied from various abiotic stresses and phytohormones treatment. The distribution of *cis-*elements in the upstream regions of promoters for the selected 15 *SrbZIP* genes was investigated. The analysis revealed that the upstream regions of promoters of the 15 *SrbZIP* genes contained abundant *cis*-elements, including photoresponsive elements, phytohormone-responsive elements and stress-responsive elements (Figure 8; Table S6). We postulated that these 15 *SrbZIP* genes were influenced by light, abiotic stress and phytohormone. To further explore whether *SrbZIP* genes have the potential to participate in stevia glycoside biosynthesis, we analyzed the gene expressions of 15 *SrbZIP* genes under different treatment conditions to assess their potentials, which could provide a reference for further exploration of the regulation mechanism of stevia glycoside synthesis pathway.

**Figure 9:**
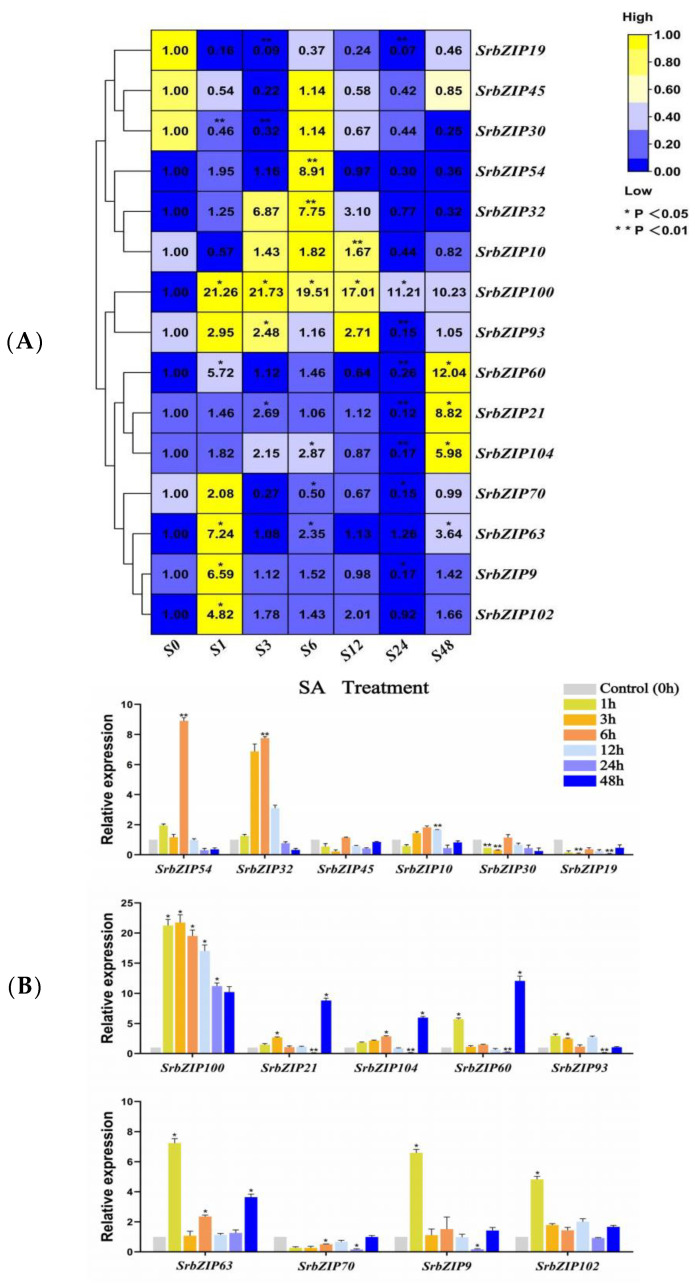
Expression patterns of *SrbZIP* genes under SA treatment analyzed by qRT-PCR. (**A**) The capital letter S indicates the SA treatment and the numbers indicate time points after treatment. The expression levels range from low expression (blue) to high expression (yellow). (**B**) A mixed-effects model (treatment effect, time effect and treatment/time interaction effect) was conducted (mixed-effects model: Time: × Treatment *p* < 0.0001). Correct for multiple comparisons using Bonferroni’s multiple comparisons test. The expression level at 0 h was set as 1. Error bars indicate SE of three biological and technical replicates, and significant differences between treatment group and control group are denoted by asterisk(s) (** *p* < 0.01, * *p* < 0.05). Different colored bars represent different treatment time points.

Phytohormones such as salicylic acid (SA) [18], methyl jasmonate (MeJA) [19] and gibberellic acid [20] enhanced the stevioside content in *S. rebaudiana* cultivated *in vitro*. Hereby, we evaluated the sensitivity of 15 *SrbZIP* genes to exogenous SA, MeJA and gibberellic acid. Twelve *SrbZIP* genes (*SrbZIP102*, *SrbZIP9*, *SrbZIP63*, *SrbZIP70*, *SrbZIP104*, *SrbZIP21*, *SrbZIP60*, *SrbZIP93*, *SrbZIP100*, *SrbZIP10*, *SrbZIP32* and *SrbZIP54*) of analyzed genes were up-regulated under SA treatment (Figure 9). *SrbZIP100* showed a substantial 21-fold increase in expression level after 1 h treatments compared with 0 h. *SrbZIP54* attained its highest expression level after 6 h, with a 9-fold increase compared with 0 h. The expression levels of 9 *SrbZIP* genes (*SrbZIP102*, *SrbZIP9*, *SrbZIP63*, *SrbZIP70*, *SrbZIP93*, *SrbZIP100*, *SrbZIP10*, *SrbZIP32* and *SrbZIP54*) firstly increased and then decreased and showed the highest expression levels after 1 and 12 h, respectively. Additionally, the expression levels of 3 *SrbZIP* genes (*SrbZIP104*, *SrbZIP21* and *SrbZIP60*) showed an increasing trend and reached the maximum after 48 h (Figure 9A,B; Table S10).

**Figure 10:**
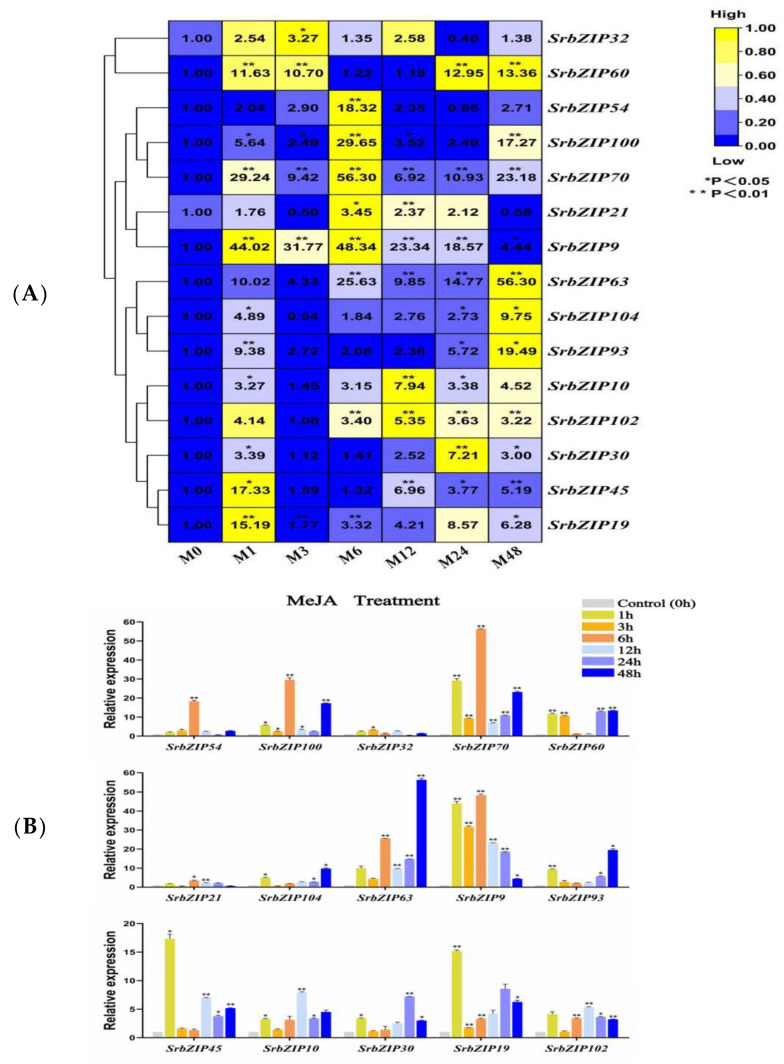
Expression patterns of *SrbZIP* genes under MeJA treatment analyzed by qRT-PCR. (**A**) The capital letter M indicates the MeJA treatment, and the numbers indicate time points after treatment. The expression levels range from low expression (blue) to high expression (yellow). (**B**) A mixed-effects model (treatment effect, time effect and treatment/time interaction effect) was conducted (mixed-effects model: Time: × Treatment *p* < 0.0001). Correct for multiple comparisons using Bonferroni’s multiple comparisons test. The expression level at 0 h was set as 1. Error bars indicate SE of three biological and technical replicates, and significant differences between treatment group and control group are denoted by asterisk(s) (** *p* < 0.01, * *p* < 0.05). Different colored bars represent different treatment time points.

The expression levels of the 15 *SrbZIP* genes in *S. rebaudiana* leaf samples sprayed with MeJA were analyzed, and the 15 *SrbZIP* genes were all affected and up-regulated by MeJA treatment (Figure 10). *SrbZIP9* and *SrbZIP63* exhibited the highest induction after MeJA stress treatment, with a 48-fold and 56-fold increase, respectively. The *SrbZIP54*, *SrbZIP100* and *SrbZIP70* genes also showed significant increases, with peak expression levels observed after 6 h treatments, resulting in 18-fold, 30-fold and 56-fold increases, respectively. The expression of 6 *SrbZIP* genes (*SrbZIP102*, *SrbZIP10*, *SrbZIP70*, *SrbZIP93*, *SrbZIP63* and *SrbZIP60*) generally remained up-regulated with the extension of treatment time, *SrbZIP93* attained the maximum (19-fold) after a 48 h treatment. Within 24 h after MeJA treatment, the expression level of *SrbZIP9* remained at a 19–48-fold increase compared with the control. Meanwhile, the expression levels of *SrbZIP45* and *SrbZIP19* genes first increased and then decreased, attaining the maximum after 1 h, resulting in 17-fold and 15-fold increases, respectively, compared with 0 h (Figure 10A,B; Table S10).

**Figure 11:**
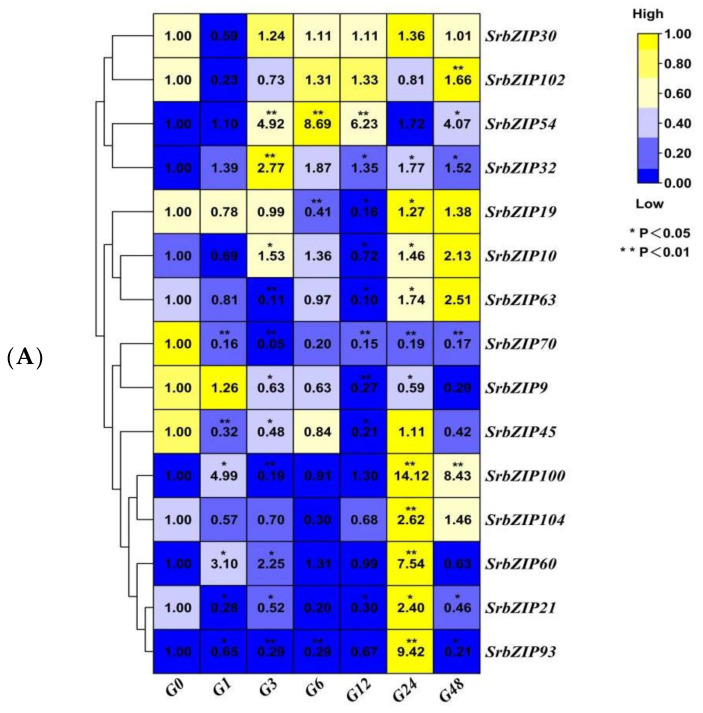

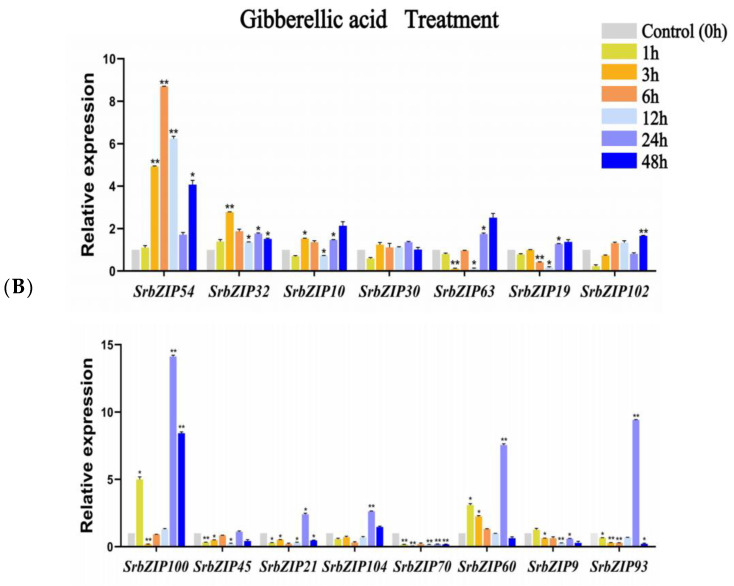
Expression patterns of *SrbZIP* genes under gibberellic acid treatment via qRT-PCR analysis. (**A**) The capital letter G indicates the gibberellic acid treatment and the numbers indicate time points after treatment. The expression levels range from low expression (blue) to high expression (yellow). (**B**) A mixed-effects model (treatment effect, time effect and treatment/time interaction effect) was conducted (mixed-effects model: Time: × Treatment *p* < 0.0001). Correct for multiple comparisons using Bonferroni’s multiple comparisons test. The expression level at 0 h was set as 1. Error bars indicate SE of three biological and technical replicates, and significant differences between treatment group and control group are denoted by asterisk(s) (** *p* < 0.01, * *p* < 0.05). Different colored bars represent different treatment time points.

The expression levels of *SrbZIP30* and *SrbZIP102* genes were initially down-regulated upon gibberellic acid treatment after 1 h and then recovered to the same level as the control (Figure 11). On the other hand, *SrbZIP54* and *SrbZIP32* genes showed moderate increases, reaching their highest expression levels after 6 h and 3 h treatments, with a 9-fold and 2-fold increase, respectively, compared with 0 h. The expression level of *SrbZIP19* and *SrbZIP63* genes began to be down-regulated after gibberellic acid treatment and showed slight up-regulation after 24 h. On the contrary, the expression level of *SrbZIP70* and *SrbZIP9* genes exhibited a down-regulated trend. *SrbZIP45*, *SrbZIP100*, *SrbZIP104*, *SrbZIP60*, *SrbZIP21* and *SrbZIP93* first decreased and then increased after gibberellic acid treatment and showed a significant decrease after 24 h treatments. Overall, the gibberellic acid treatment showed negative regulation to most of these 15 *SrbZIP* genes (Figure 11A,B; Table S10).

Previous research indicates that a long-day (16 h) photoperiod significantly increased *S. rebaudiana* leaf biomass and stevia glycoside (SGs) content [36]. Moreover, under varying light intensities, the biosynthetic genes of stevia glycoside exhibited differential expressions, leading to changes in stevioside (ST) and rebaudioside A (RA) contents [37]. Additionally, the maximum biomass accumulation in the callus culture of *S. rebaudiana* was induced by white light compared with yellow, blue, green and red lights. We further explored the effect of the light on the 15 *SrbZIP* genes by treating stevia leaves with white light and dark.

**Figure 12:**
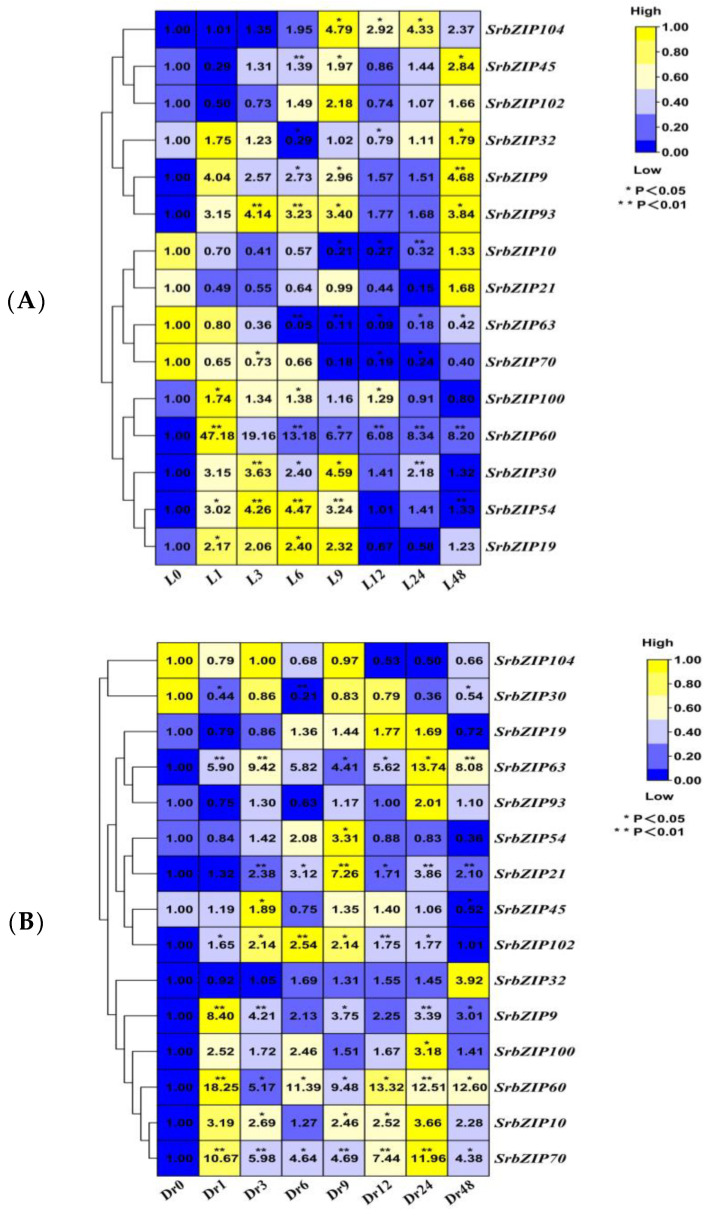

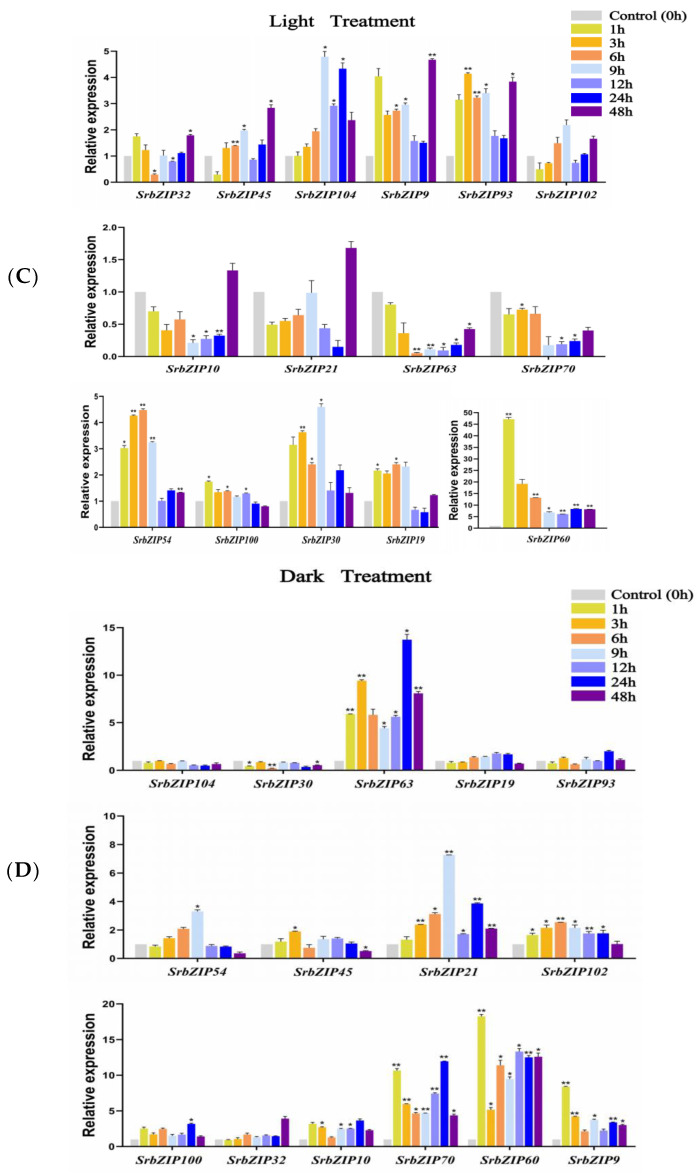
Expression patterns of *SrbZIP* genes under light and dark treatment via qRT-PCR analysis. (**A**) The capital letter “L” indicates the light treatment, and the numbers indicate time points after treatment. (**B**) The “Dr ”indicates the dark treatment, and the numbers indicate time points after treatment. The expression levels range from low expression (blue) to high expression (yellow). (**C**) Expression patterns of *SrbZIP* genes under light treatment via qRT-PCR analysis. (**D**) Expression patterns of *SrbZIP* genes under dark treatment via qRT-PCR analysis. A mixed-effects model (treatment effect, time effect and treatment/time interaction effect) was conducted (mixed-effects model: Time: × Treatment *p* < 0.0001). Correct for multiple comparisons using Bonferroni’s multiple comparisons test. The expression level at 0 h was set as 1. Error bars indicate SE of three biological and technical replicates, and significant differences between treatment group and control group are denoted by asterisk(s) (** *p* < 0.01, * *p* < 0.05). Different colored bars represent different treatment time points.

Except for *SrbZIP70*, *SrbZIP63*, *SrbZIP21* and *SrbZIP10* genes, other 11 *SrbZIP* genes were up-regulated to varying degrees after light treatment (Figure 12A). The *SrbZIP60* gene was rapidly induced and reached the maximum expression after 1 h light treatment, followed by a decline, but it remained up-regulated compared with the control. The expression levels of *SrbZIP30*, *SrbZIP54* and *SrbZIP19* genes firstly increased and then decreased, reaching the maximum after 9 h and 6 h light treatment, resulting in 5-fold, 4-fold and 2-fold increases, respectively, compared with the control. Likewise, *SrbZIP93*, *SrbZIP9* and *SrbZIP32* genes displayed an initial up-regulation followed by down-regulation after light treatment, with down-regulation observed after a 12 h treatment, then the expression levels increased slightly, indicating that these genes may be influenced by circadian rhythms. On the contrary, *SrbZIP45* and *SrbZIP102* genes first decreased and then increased after light treatment. In the early stage of light treatment, light negatively regulated the expression of the two *SrbZIP* genes. When the stevia leaves were shaded, some *SrbZIP* genes showed the opposite variation trend (Figure 12B). *SrbZIP70*, *SrbZIP10*, *SrbZIP100*, *SrbZIP9*, *SrbZIP60* and *SrbZIP63* genes were rapidly induced upon dark treatment, with the extension of treatment time, the up-regulated trend was maintained compared with 0 h, *SrbZIP70* and *SrbZIP10* genes showed highest expression with a 12-fold and 4-fold after 24 h dark treatment. Interestingly, whether the *SrbZIP60* gene was treated with light or dark, it was rapidly induced and maintained highly expressed after a 1 h treatment. The expression levels of *SrbZIP104* and *SrbZIP30* continued to decline, while *SrbZIP32* continued to increase. *SrbZIP19*, *SrbZIP93* and *SrbZIP54* genes first decreased and then increased after dark treatment and showed a significant decrease after 24 h treatments. Dark treatment negatively regulates the expression of these *SrbZIP* genes, which may trigger the defense mechanisms of stevia. It is also related to the circadian rhythm (Figure 12C,D; Table S10).

**Figure 13:**
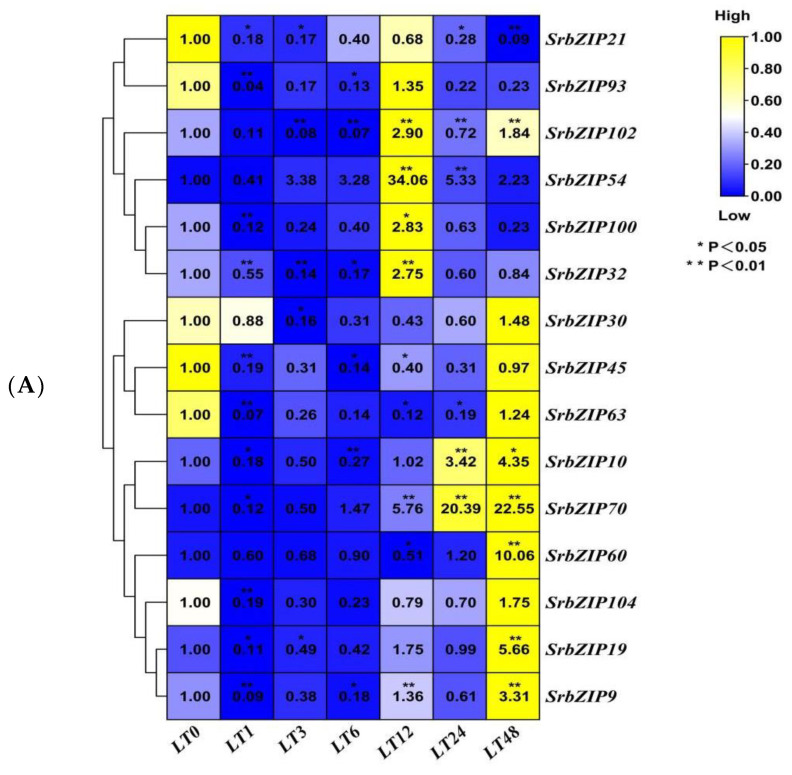

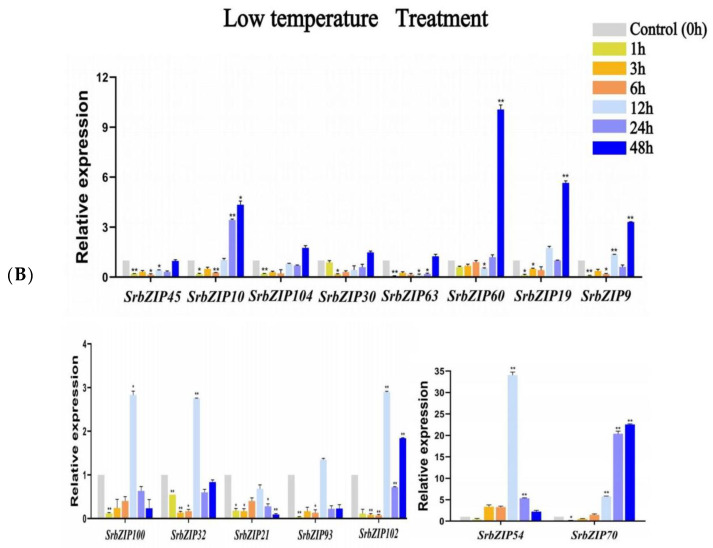
Expression patterns of *SrbZIP* genes under low temperature (4 °C) treatment via qRT-PCR analysis. (**A**) The “LT” indicates the low temperature (4 °C) treatment, and the numbers indicate time points after treatment. The expression levels range from low expression (blue) to high expression (yellow). (**B**) A mixed-effects model (treatment effect, time effect and treatment/time interaction effect) was conducted (mixed-effects model: Time: × Treatment *p* < 0.0001). Correct for multiple comparisons using Bonferroni’s multiple comparisons test. The expression level at 0 h was set as 1. Error bars indicate SE of three biological and technical replicates, and significant differences between treatment group and control group are denoted by asterisk(s) (** *p* < 0.01, * *p* < 0.05). Different colored bars represent different treatment time points.

Inappropriate temperature conditions inhibit plant growth and development, which strongly influences secondary metabolism accumulation. Additionally, a study has shown that all of the fifteen genes of the stevia glycoside (SGs) biosynthesis pathway were transcribed maximally at 25 °C, while both low (15 °C) and high temperatures (35 °C) restrained their transcription [21]. As shown in Figure 13, the expression levels of all 15 *SrbZIP* genes significantly down-regulated within a short time frame (1–3 h). *SrbZIP93*, *SrbZIP102*, *SrbZIP54*, *SrbZIP100* and *SrbZIP32* genes have shown the highest expression levels after a 12 h low-temperature treatment, subsequently decreased. Within a period of time, these genes may play a crucial role in the tolerance to low-temperature stress in *S. rebaudiana*. Notably, the *SrbZIP54* gene was dramatically increased and reached the maximum expression level after a 12 h treatment, with a 34-fold increase compared with the control. Even when down-regulated, its expression level remained higher than the control. The expression levels of the *SrbZIP21* and *SrbZIP45* genes were continuously down-regulated. The *SrbZIP30*, *SrbZIP63*, *SrbZIP10*, *SrbZIP70*, *SrbZIP60*, *SrbZIP104*, *SrbZIP19* and *SrbZIP9* genes firstly down-regulated and then up-regulated, reached maximum expression after a 48 h low-temperature treatment. Different *SrbZIP* genes played distinct physiological regulatory roles at different stress durations (Figure 13A,B; Table S10).

**Figure 14:**
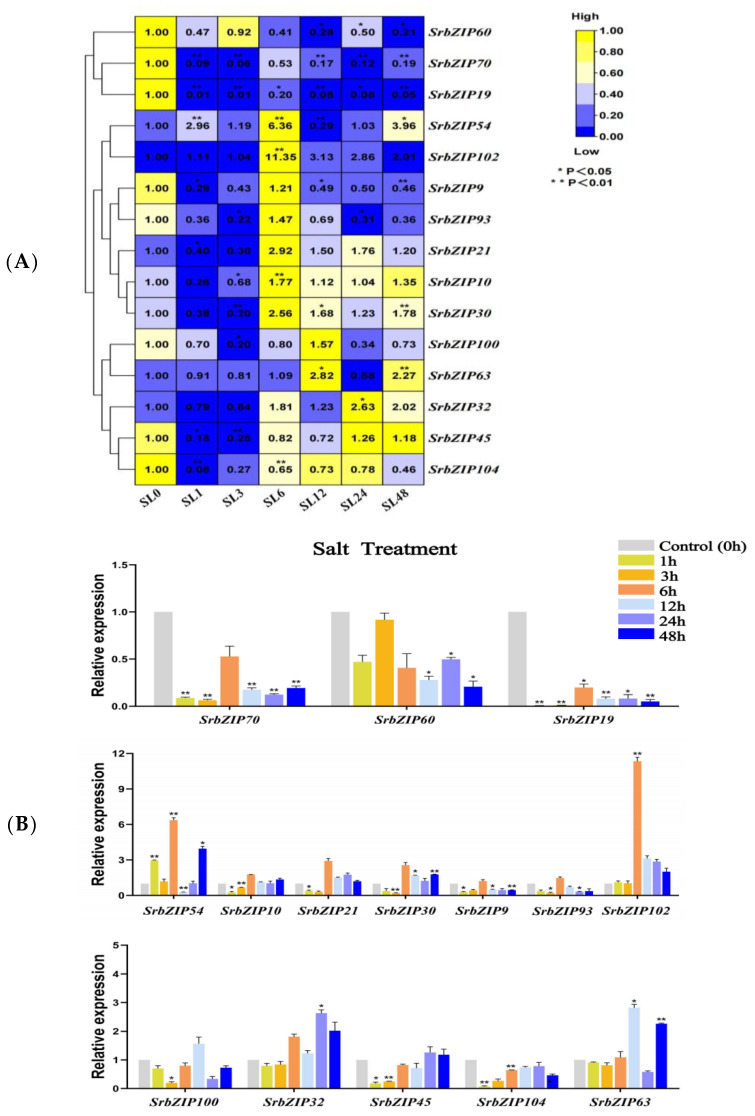
Expression patterns of *SrbZIP* genes under salt stress via qRT-PCR analysis. (**A**) The “SL” indicates the salt stress and the numbers indicate time points after salt treatment. The expression levels range from low expression (blue) to high expression (yellow). (**B**) A mixed-effects model (treatment effect, time effect and treatment/time interaction effect) was conducted (mixed-effects model: Time: × Treatment *p* < 0.0001). Correct for multiple comparisons using Bonferroni’s multiple comparisons test. The expression level at 0 h was set as 1. Error bars indicate SE of three biological and technical replicates, and significant differences between treatment group and control group are denoted by asterisk(s) (** *p* < 0.01, * *p* < 0.05). Different colored bars represent different treatment time points.

It was found that salt stress (NaCl) promoted the accumulation of stevia glycoside (SGs) significantly [16] and up-regulated several stevia glycoside (SGs) biosynthesis pathway genes (*CMS*, *CMK*, *HDR* and *UGT76G1*) [38]. To investigate the expression pattern of the 15 *SrbZIP* genes in stevia leaves under abiotic stress induced by the salts for different durations, we constructed heatmaps to evaluate their responsiveness to salt stress (Figure 14). Evidently, ten genes (*SrbZIP60*, *SrbZIP70*, *SrbZIP19*, *SrbZIP9*, *SrbZIP93*, *SrbZIP21*, *SrbZIP10*, *SrbZIP30*, *SrbZIP45* and *SrbZIP104*) were significantly down-regulated after a 1 h salt treatment. *SrbZIP60*, *SrbZIP70* and *SrbZIP19* genes were continuously down-regulated after salt stress. Following salt treatment for 6 h, two genes (*SrbZIP54* and *SrbZIP102*) were significantly up-regulated by approximately 6 and 11 folds, respectively, and then decreased. These positively regulated *SrbZIP* genes under salt stress may be conducive to *S. rebaudiana* improving its tolerance to abiotic stress and promoting plant growth and development. The expression of five genes (*SrbZIP9*, *SrbZIP93*, *SrbZIP21*, *SrbZIP10* and *SrbZIP30*) first decreased and then increased, showing the highest expression upon a 6 h salt treatment. *SrbZIP104* gene was significantly down-regulated and then slightly recovered expression but lower than the control, while *SrbZIP45* gradually reached the 0 h expression level. In summary, most *SrbZIP* genes showed down-regulated expression following early salt treatment, while a few *SrbZIP* genes showed a slightly delayed response process (Figure 14A,B; Table S10).

**Figure 15:**
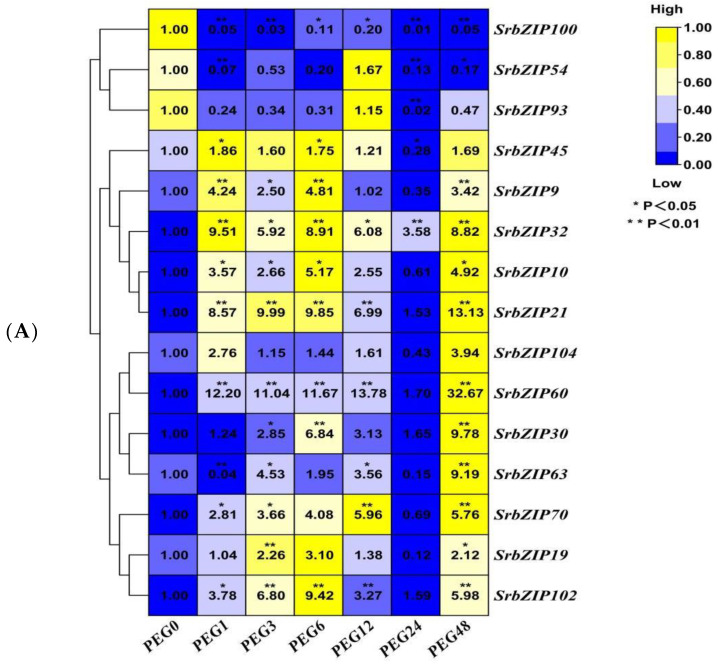

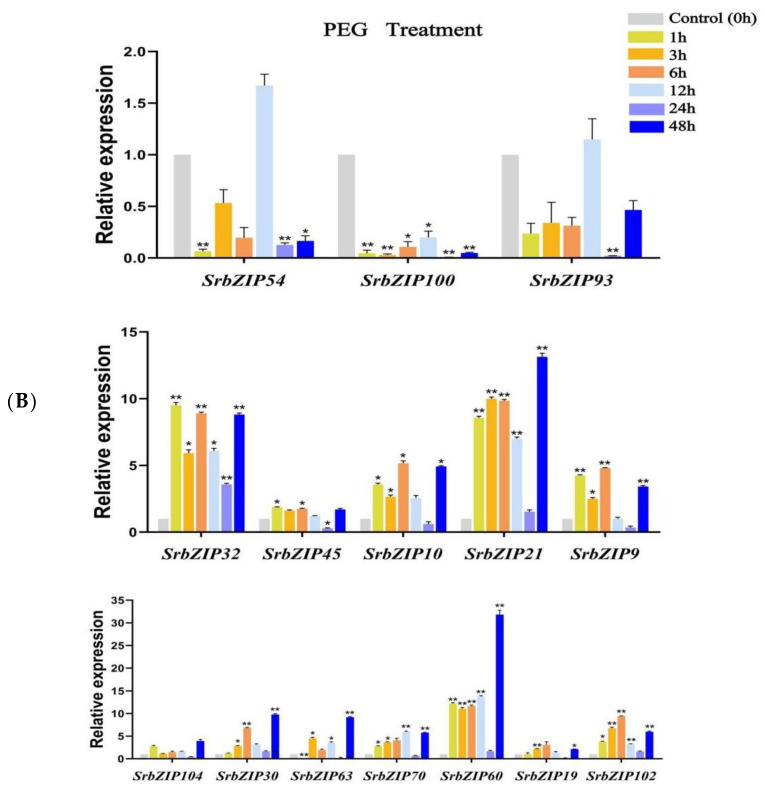
Expression patterns of *SrbZIP* genes under PEG treatment via qRT-PCR analysis. (**A**) The “PEG” indicates the 5% PEG4000 treatment, and the numbers indicate time points after salt treatment. The expression levels range from low expression (blue) to high expression (yellow). (**B**) A mixed-effects model (treatment effect, time effect and treatment/time interaction effect) was conducted (mixed-effects model: Time: × Treatment *p* < 0.0001). Correct for multiple comparisons using Bonferroni’s multiple comparisons test. The expression level at 0 h was set as 1. Error bars indicate SE of three biological and technical replicates, and significant differences between treatment group and control group are denoted by asterisk(s) (** *p* < 0.01, * *p* < 0.05). Different colored bars represent different treatment time points.

A study has shown that the production of steviol glycosides (SGs) in the callus and suspension culture of *S. rebaudiana* treated with polyethylene glycol (PEG) was enhanced [17]. The 15 *SrbZIP* genes showed different expression patterns under drought stresses caused by PEG treatment (Figure 15). With the prolonged stress time, the expression level of 10 *SrbZIP* genes (*SrbZIP21*, *SrbZIP10*, *SrbZIP32*, *SrbZIP9*, *SrbZIP45*, *SrbZIP102*, *SrbZIP19*, *SrbZIP70*, *SrbZIP60* and *SrbZIP104*) showed an initial increase, followed by a decline, and reached significantly lower levels after a 24 h treatment, finally increased again after a 48 h treatment. The expression levels of *SrbZIP21*, *SrbZIP63*, *SrbZIP30*, *SrbZIP60* and *SrbZIP104* genes showed the highest expression after 48 h, with a 13-fold, 9-fold, 10-fold, 33-fold and 4-fold increase, respectively, compared with the control. *SrbZIP60* exhibited a rapid response to drought stress and maintained a higher expression level than the control, except for 24 h. After a 24 h treatment, other factors may come into play, with *SrbZIP* genes working in concert with other genes, enhancing drought tolerance. The expression levels of *SrbZIP100*, *SrbZIP54* and *SrbZIP93* generally down-regulated after PEG treatment. In summary, most *SrbZIP* genes showed a fluctuating state for responding to drought stress, while a few *SrbZIP* genes were negatively regulated by the drought stress (Figure 15A,B; Table S10).

After MeJA treatment, 12 *SrbZIP* genes (*SrbZIP102*, *SrbZIP9*, *SrbZIP63*, *SrbZIP70*, *SrbZIP104*, *SrbZIP21*, *SrbZIP60*, *SrbZIP93*, *SrbZIP100*, *SrbZIP10*, *SrbZIP32* and *SrbZIP54*) were up-regulated upon SA treatment, while another 3 *SrbZIP* genes were down-regulated under SA stress condition. As for MeJA treatment, all 15 *SrbZIP* genes were affected and up-regulated, and the speed of response to MeJA was different. *SrbZIP70* and *SrbZIP9* genes were dramatically increased and reached the maximum expression levels after 6 h post-MeJA treatment. The gibberellic acid treatment showed negative regulation to most genes of the 15 *SrbZIP* genes, but *SrbZIP54*, *SrbZIP100* and *SrbZIP60* genes were modestly increased and highly expressed upon 6 h and 24 h treatments, with 9-fold, 14-fold and 8-fold increases, respectively. Except for *SrbZIP70*, *SrbZIP63*, *SrbZIP21* and *SrbZIP10* genes, other 11 *SrbZIP* genes were up-regulated to varying degrees after light treatment, and *SrbZIP54*, *SrbZIP30*, *SrbZIP60*, *SrbZIP93* and *SrbZIP9* genes were rapidly induced. Among them, whether the *SrbZIP60* gene was treated with light or dark, it was rapidly induced and maintained a higher expression level. Additionally, *SrbZIP54*, *SrbZIP70*, *SrbZIP60* and *SrbZIP19* were up-regulated from the control at the highest expression levels with approximately 34 folds, 23 folds, 10 folds and 6 folds, respectively, after low-temperature treatment. After a 6 h salt treatment, two genes (*SrbZIP54* and *SrbZIP102*) were dramatically up-regulated with a 6-fold and 11-fold, respectively, compared with 0 h. With prolonged salt stress, the expression level of 10 *SrbZIP* genes (*SrbZIP104*, *SrbZIP60*, *SrbZIP70*, *SrbZIP30*, *SrbZIP19*, *SrbZIP102*, *SrbZIP9*, *SrbZIP32*, *SrbZIP10* and *SrbZIP21*) showed a total trend of increased, except for a 24 h treatment, and *SrbZIP60* extremely fast response to drought stress. These results elucidated the different responsive mechanisms of *SrbZIPs* under light treatment, phytohormones treatment and abiotic stresses. The characteristics of *SrbZIPs* can be more effectively explored to tap their potential in the future.

### 3.9. Analysis of Terpenoid Synthesis-Related SrbZIP Genes That Respond to Light Treatment, Phytohormones Treatment and Abiotic Stresses

**Figure 16:**
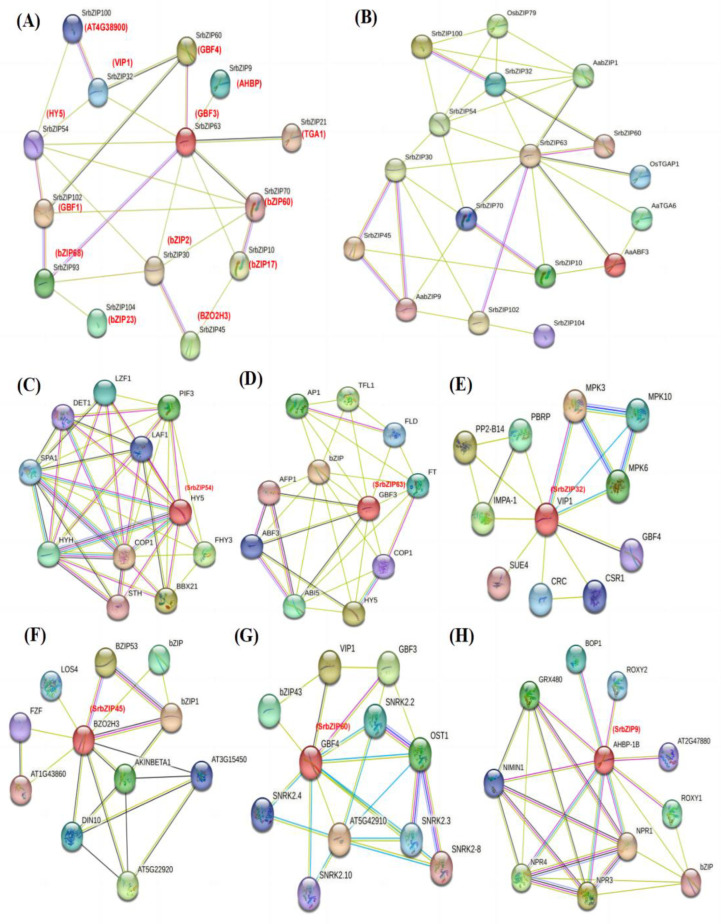
Protein interaction network analysis. (**A**) Predicted protein–protein interaction network of SrbZIP protein. (**B**) Predicted protein–protein interaction network between SrbZIP proteins and reported bZIP protein involved in terpenoid synthesis. The network nodes represent proteins, and the line colors indicate the types of evidence (purple lines: experimentally determined; black lines: co-expression; lilac lines: protein homology; green lines: text mining). (**C**) Analysis of SrbZIP54 protein interaction network. (**D**) Analysis of SrbZIP63 protein interaction network. (**E**) Analysis of SrbZIP32 protein interaction network. (**F**) Analysis of SrbZIP45 protein interaction network. (**G**) Analysis of SrbZIP60 protein interaction network. (**H**) Analysis of SrbZIP9 protein interaction network.

To investigate the potential *SrbZIP* genes associated with steviol glycosides (SGs) biosynthesis and focused on specific genes, we utilized the STRING software (v12.0) to analyze the protein interaction network between the 15 SrbZIP proteins and their homologous AtbZIP proteins in *A. thaliana* (Figure 16A; Table S9). Additionally, by searching relevant references, we selected candidate related proteins involved terpenoid synthesis, including AabZIP1 (GenBank: PWA69369.1) [39], AabZIP9 (GenBank: MG584701) [40], AaTGA6 (GenBank: MH201467) [41] and AaABF3 (GenBank: MH734935) [42], OsbZIP79 (Os11g0152700) [43] and OsTGAP1 (Os04g0637000) [44] (Figure 16B). We constructed a protein interaction network (*p*-value: <1.0 × 10^−16^) relationship map with 14 nodes and 25 edges (Figure 16A). It was found that there were 6 members (SrbZIP54 (HY5), SrbZIP60 (GBF4), SrbZIP9 (AHBP), SrbZIP70 (bZIP60), SrbZIP30 (bZIP2) and SrbZIP10 (bZIP17)) were predicted to be involved in positive regulation of metabolic process (GO: 0009893), of which 3 members (SrbZIP70, SrbZIP10 and SrbZIP9) and other 2 members (SrbZIP32 (VIP1) and SrbZIP45 (BZO2H3)) related to the cellular response to stress (GO:0033554). Through the prediction of protein–protein interaction between SrbZIP proteins and 6 reported bZIP proteins involved in terpenoid synthesis, it was found that SrbZIP54 (HY5), SrbZIP63 (GBF3), SrbZIP32 (VIP1) and SrbZIP45 (BZO2H3) had interaction with terpenoid synthesis-related bZIP proteins (Figure 16B). SrbZIP54 (HY5) was predicted to interact with other proteins (COP1, PIF3, PHY and SPA1), functioning as the center of a transcriptional network hub and a master regulator of light signal (Figure 16C). SrbZIP63 (GBF3) was predicted to be associated with most terpenoid synthesis-related proteins, indicating its potential involvement in terpenoid synthesis. Furthermore, SrbZIP63 (GBF3) was predicted to interact with abscisic acid-responsive element binding factor 3 (ABF3)/abscisic acid insensitive (ABI5) participating in ABA signaling pathway with FT and AP1, which is involved in the regulation of flowering, and with HY5, which specifically bind to G-box (Figure 16D). SrbZIP32 (VIP1) was predicted to interact with (mitogen-activated protein kinases) MPKs (Figure 16E), which are induced by stress, cytokines, plant hormones and growth factors and participated in plant signaling [45]. The results showed that the potential function of SrbZIP32 (VIP1) participated in responding to stress and resisting disease. SrbZIP45 (BZO2H3) was predicted to interact with AtbZIP53 and AtbZIP1 (Figure 16F), which were pivotal regulatory factors involved in energy deficiency, sucrose starvation, and senescence-induced nutrient translocation [46] and with AKINBETA1 which involved in regulating nitrogen and sugar metabolism [47]. In addition, based on the expression and response speed of 15 SrbZIPs after various treatments, we selected two other SrbZIP proteins (SrbZIP60 and SrbZIP9) to construct a protein–protein interaction network. SrbZIP60 (GBF4) was predicted to interact with (sucrose non-fermenting-1-related protein kinase) SNRK (Figure 16G), which was involved in different stress signal transduction pathways and participated in resisting adverse environments [48], and with (open stomata 1) OST1 which involved in resisting to low-temperature stress [49]. SrbZIP9 (AHBP-1B) was predicted to interact with (Arabidopsis nonexpresser of PR genes) NPRs (Figure 16H), which were positively regulated SA-dependent signaling pathways while negatively regulated JA-dependent signaling pathways [50,51]. These results suggest that SrbZIP60 (GBF4) and SrbZIP9 (AHBP-1B) may play a joint role in tolerance to abiotic stress.

## 4. Discussion

### 4.1. Classification and Gene Duplication of SrbZIPs

*S. rebaudiana* is a perennial herb renowned for its high sweetness and low-calorie characteristics. Its leaves contain natural sweetener steviol glycosides (SGs) with numerous health benefits, which is a tetracyclic diterpenoid derivative. The plant bZIP transcription factors have proved to be involved in multiple biological processes, such as tissue and organ development, responses to abiotic and biotic stress and secondary metabolism [52]. Although there have been reports of studies sequencing the stevia genome [15], bZIP family genes in *S. rebaudiana* have not been comprehensively identified, and their roles in terpenoid synthesis are unclear.

A total of 105 *SrbZIP* genes were discovered in the *S. rebaudiana* genome by a homology search in this study. Compared with other reported species, such as *Arabidopsis* (78) [1], rice (*Oryza sativa*) (89) [53], sorghum (92) [54], soybean (*Glycine max*) (160) [55], poplar (86) [2], licorice (*G. uralensis*) (66) [4], safflower (*C. tinctorius*) (52) [5] and Marijuana (*Cannabis sativa*) (58) [56], *S. rebaudiana* had the second highest number of bZIP genes. The phylogenetic relationship of *SrbZIPs* showed that bZIP family genes in *S. rebaudiana* were classified into 12 subgroups, which were similarly observed in those of *C. tinctorius* [5]. The *SrbZIP* genes classified in subgroup S contained the most members, while subgroups J and K contained the fewest members. However, *SrbZIP77* could not be aggregated into any subfamily. We speculated that the structure of the *SrbZIP77* has been greatly different in evolution or differed from the evolutionary direction of the *A. thaliana* bZIP family.

Additionally, we observed that the 105 *SrbZIP* genes were distributed across 11 chromosomes in *S. rebaudiana* with no apparent pattern (Figure 2A). *SrbZIP* genes distributed on chromosome 2 shared the biggest number of members (21). This uneven distribution may be attributed to differences in chromosome size and structure. The variation in the bZIP gene family among different plant species could be influenced by the genome size or the gene duplication events during evolution [57]. Gene duplication generally occurs through two major mechanisms: segmental and tandem duplications, representing distinct evolutionary patterns in plants [58]. Chromosome rearrangement generates numerous duplicated chromosomal blocks that result in segmental duplication events occurring [58]. It has been revealed that segmental duplication during evolution resulted in the expansion of various gene families [26]. In our study, the results suggested that the expansion of the bZIP gene family in *S. rebaudiana* was mainly attributed to segmental duplication. Twenty-seven pairs of gene segmental duplications were detected on eleven chromosomes and one pair (*SrbZIP67* and *SrbZIP68*) with evidence for tandem duplication (Figure 2A; Table S4). Furthermore, there was great collinearity between *S. rebaudiana* and *A. thaliana*, with sixty-two orthologous pairs identified to exist on all the chromosomes (Figure 2B; Table S5).

### 4.2. Structure Characteristic and Function Prediction of SrbZIPs

The *SrbZIP* gene family exhibited high conservation, with all members sharing characteristic motif 1 regions, and the conserved motifs within the same subfamily were closely related to the gene structure (Figure 3). Ten motifs were identified in the *SrbZIP* gene family through the motif analysis, named motif 1 to motif 10 (Figure S1). The overall compositions of motifs were similar within the same subfamily. The numbers and lengths of exons varied considerably among different *SrbZIP* subfamilies, resulting in gene lengths ranging from 96 to 704 amino acids (Figure 3C; Table S2). In eukaryotes, the variation in intron locations and numbers may account for some specific gene functions and evolutionary trajectories, and intron evolution is often associated with gene segmental duplication [59]. Our study revealed that 19% of the total *SrbZIP* genes were intronless (Figure 3C), which were similar to those of rice (15.3%) [53], tomato (17.4%) [60] and poplar (22%) [2]. Subgroup S contained 25 members of *SrbZIP* genes. Among them, 18 members of the S subgroup were intronless. Similarly, 19 members were classified in the S subgroup in poplar, with 18 members containing no introns [2]. These results indicated that the gene structures of *SrbZIP* genes that belonged to the S subgroup were highly conserved across different species.

*Cis*-elements are essential for the transcriptional regulation of gene expression in response to abiotic stresses [61]. Photoresponsive elements, followed by ABA, MeJA, gibberellin and SA responsive elements, were among the major *cis*-elements in the *SrbZIP* promoter regions (Figure 4). In addition, the defense and stress-responsive elements (low-temperature, salt and drought stress, etc.) were frequently discovered in *SrbZIP* promoters. These *cis*-regulatory elements in *SrbZIP* promoters provided foundational evidence for the functional relevance of *SrbZIP* genes in regulating the growth and response to various abiotic/biotic stresses.

Proteins interact with each other to participate in intracellular/intercellular signal transduction energy and material metabolism, regulation of gene expression, and regulation of cell cycle. Through the homology search of AtbZIPs, some orthologous pairs were identified to exist between SrbZIPs and AtbZIPs, which revealed the potential functional relevance of most SrbZIP proteins. To further tap their potential, we carried out an interaction prediction network analysis utilizing the STRING database (v12.0), searching multiple sequences (Figure 5). SrbZIP proteins were mainly classified into four major categories: involved in phytohormone signaling, involved in environmental signaling, involved in cryptochrome signaling and promoted flowering.

### 4.3. Expression Patterns of SrbZIP Genes and Light, Phytohormone and Abiotic Stress Response

Each *SrbZIP* gene’s profile of expression in six *S. rebaudiana* cultivars was elucidated. The *SrbZIP* genes also verified the differential expression among stevia cultivars. Comparative DEG analysis revealed that 5 bZIP genes (*SrbZIP45*, *SrbZIP93*, *SrbZIP102*, *SrbZIP19* and *SrbZIP104*) (*p* < 0.01) significantly differed between the ‘023’ cultivar bZIP genes and other 5 cultivars. In addition, 10 *SrbZIP* genes from different bZIP subfamilies expressed higher of the‘023’ *S. rebaudiana* cultivar (Figure 6). Most genes of the 15 *SrbZIP* members were differently expressed among various tissues (Figure 7). *SrbZIP63*, *SrbZIP54* and *SrbZIP70* showed higher expression levels in leaves, which may be related to leaf development. *SrbZIP30* presented high expression in flowers, which may relate to the regulation of flowering.

We found that stevia glycosides varied among various abiotic stresses and phytohormone treatments [35]. The expression of 15 *SrbZIP* genes under different treatment conditions was analyzed, which provided a reference for further exploration of the regulation mechanism of the stevia glycoside synthesis pathway. Except for drought stress and dark treatment, *SrbZIP54* exhibited up-regulation in all phytohormone treatments and abiotic stresses, with expression levels consistently higher than the control (Figure S3), attributed to the abundant *cis*-elements in the upstream regions of promoters of *SrbZIP54* gene in *S. rebaudiana*, including stress response, phytohormone response and light response elements (Figure 8). SrbZIP54 (HY5) was predicted to interact with other proteins (PIF3, COP1, PHY and SPA1), suggesting it functions as a central transcriptional network hub and a master regulator of light signaling (Figure 16C). ELONGATED HYPOCOTYL5 (*HY5*) inhibits hypocotyl growth and lateral root development and promotes pigment accumulation in a light-dependent manner in *A. thaliana* [61]*. HY5* is a major regulator regulating plant growth and development, including cell elongation and proliferation, chloroplast development, pigment accumulation and nutrient assimilation [62,63]. Recently, the role of *HY5* in other aspects has also been reported, such as hormone signal transduction, plant defense and temperature response [64,65]. Most importantly, *HY5* also regulated the terpenoid synthesis, reported in *Artemisia* (*AaHY5*) [12] and in *A. thaliana* (*AtHY5*) [66]. It provided evidence that *HY5* may act as a potential transcriptional regulator for stevia biosynthesis.

*SrbZIP63* (*GBF3*) was up-regulated in drought treatments (Figure 15). The result can also be verified in *A. thaliana*. Overexpression of *AtGBF3* in *A. thaliana* enhanced the tolerance to osmotic stress, salinity and drought stress [67]. *SrbZIP63* was up-regulated after dark treatment and, on the contrary, was down-regulated after light treatment, which is consistent with the results reported in the study of *GBF3* mRNA predominantly in dark-grown leaves and in roots [68]. In the same subfamily (G-subfamily), *SrbZIP93* and *SrbZIP102* showed different expression trends after light treatment, with *SrbZIP102* preferring dark treatment. Meanwhile, *SrbZIP102* and *SrbZIP63* showed the same expression trends after phytohormone treatment (SA, MeJA and GA). It indicated that bZIP genes from the same subfamily showed similar functions with similar structures. *SrbZIP63* (*GBF3*) was also predicted to have a potential function in terpenoid synthesis, abscisic acid response and regulation of flowering (Figure 16D).

*SrbZIP32 (VIP1)* was up-regulated with approximately 10-fold higher expression under drought stress compared with the control (Figure 15). It was predicted that it interacted with mitogen-activated protein kinase 3 (MPK3) (Figure 16E), and indeed it is. VIP1 protein not only participates in agrobacterium-mediated plant transformation but is also related to plant immune signal transduction pathways with phosphorylated by MPK3 [69]. The I-subfamily bZIP genes in *A. thaliana* reported the potential value of disease resistance and stress resistance [1]. Although only a subset of I-subfamily bZIP genes has been analyzed yet, it provides insight into the potential functional relevance of I-subfamily bZIPs to defense and stress response, regulation of cell cycle and various developmental aspects.

SrbZIP45 (BZO2H3) protein was predicted to interact with AabZIP9 (Figure 16B); however, it has a lackluster response to most stress treatments, with the expression level being down-regulated under treatments. Above all, *SrbZIP54*, *SrbZIP63* and *SrbZIP32* showed promise as potential candidates for enhancing abiotic stress tolerance and secondary metabolite production in *S. rebaudiana* through genetic improvement.

A long-day (16 h) photoperiod and high light intensity significantly increased stevia glycoside (SGs) content [36,37]. (*SrbZIP54*) *HY5* and *SrbZIP63* (*GBF3*) may function antagonistically to the response to optical signals (Figure 12A,B). *SrbZIP63* was up-regulated after dark treatment. On the contrary, it was down-regulated after light treatment, which is consistent with the results reported in the study of *GBF3* mRNA predominantly in dark-grown leaves and roots [68]. *HY5* functions as a positive regulator of light signaling; the expression of *HY5* gene light is positively regulated by light treatment [61]. However, there is no evidence of *GBF3* and *HY5* involved in the regulation of light-induced terpenoid synthesis in *S. rebaudiana.* We speculate that the terpenoid synthesis in *S. rebaudiana* includes the participation of many various factors. Overexpression of *AtGBF3* increased the expression of *AFP* (ABI five binding protein) genes and increased resistance to ABA [67]. On the contrary, *HY5* can bind to the promoter of ABA INSENSITIVE 5 (ABI5) as an important hub in the crosstalk between light and cold response pathways [70]. Abscisic acid (ABA) and gibberellic acid (GA) antagonistically regulate many developmental processes and responses to biotic or abiotic stresses in higher plants. Environmental signals such as cold and light trigger seeds to break dormancy by flipping the balance towards GA [71]. ABA is involved in suppressing GA biosynthesis. ABI5 regulates the expression of genes associated with ABA and GA metabolism and signaling to control ABA and GA levels [72]. SGs are diterpene secondary metabolites and share their biosynthesis pathway with GAs [73]. *Yoneda* et al. ascribed that the ent-kaurenoic acid (ent-KA) precursor tends toward the production of SGs by inhibiting gibberellin biosynthesis [74]. *GBF3* and *HY5* may indirectly regulate the content of GA by regulating the expression of *ABI5* in *S. rebaudiana*, thus affecting the content of SGs.

Low temperature and low-R/FR (red light (R), far-red light (FR)) conditions increased the accumulation of the SlHY5 [75], SlPIF4 [76] and MPK3 [77,78] proteins. In addition, *SlPIF4* enhances cold tolerance in tomato plants by inducing ABA and repressing GA biosynthesis. VIP1 protein is related to plant immune signal transduction pathways with phosphorylated by MPK3 [69]. MAPKs are involved in phytochrome signal transduction [79]. SrbZIP32 (VIP1) was predicted to interact with OsbZIP79, AabZIP1 and SrbZIP54 (HY5) proteins. HY5 integrates temperature, light, and hormone signaling to balance plant growth and cold tolerance [75]. Based on the factors that affect the synthesis of stevia glycosides [35], we found that stevia glycosides varied from various abiotic stresses and phytohormones treatment. To further explore the function and regulatory role of the bZIP gene family in terpenoid synthesis in *S. rebaudiana*, necessitating urgent efforts to establish a regulatory network (including environmental factors, signaling pathways and metabolic regulation).

## 5. Conclusions

Based on the complete genome data of *S. rebaudiana*, a total of 105 *SrbZIP* genes were aggregated into 12 subfamilies by the bioinformatics method. Conserved domains, exons, introns and motifs indicated similarities within bZIP clusters. Segmental duplications were predominately responsible for the expansion of the *SrbZIP* gene family. The *cis*-element analysis evaluated the potential multiple roles of *SrbZIPs* in light, phytohormone treatment and abiotic stresses. Utilizing the RNA-seq data from leaves of six *S. rebaudiana* cultivars, real-time fluorescence PCR data of *SrbZIP* gene family in different tissues and expression patterns of the selected 15 *SrbZIP* genes in response to light, phytohormones and abiotic stresses, 6 *SrbZIP* (*SrbZIP54*, *SrbZIP63*, *SrbZIP32*, *SrbZIP45*, *SrbZIP60* and *SrbZIP9*) genes were further screened and 3 *SrbZIP* genes (*SrbZIP54*, *SrbZIP63* and *SrbZIP32*) were finally identified as highly induced by one or more of these factors, potentially influencing stress-responsive terpenoid synthesis in *S. rebaudiana*. The protein–protein interaction network further expands the potential functions of *SrbZIP* genes. Our results paved the way for future functional studies to explore the roles of *SrbZIP* genes in stress-responsive terpenoid synthesis in *S. rebaudiana*.

## Data Availability

All the data in this study are included in this published article.

## Supplementary Materials

The following supporting information can be downloaded at: https://www.mdpi.com/article/10.3390/genes14101918/s1, Figure S1: Protein motifs in the bZIP members. Figure S2: Phylogenetic tree and protein domain. Figure S3: Expression patterns of *SrbZIP54* gene in response to salt, PEG, light, dark, SA, MeJA, gibberellic acid and low-temperature treatment. Figure S4: Expression patterns of *SrbZIP63* gene in response to salt, PEG, light, dark, SA, MeJA, gibberellic acid and low-temperature treatment. Figure S5: Expression patterns of *SrbZIP32* gene in response to salt, PEG, light, dark, SA, MeJA, gibberellic acid and low-temperature treatment. Table S1: Primers involved in the bZIP gene family. Table S2: Overview of bZIP TFs in *S. rebaudiana*. Table S3: Chromosomal location and fragment duplication of *SrbZIP* gene in *S. rebaudiana*. Table S4: Distribution type and chromosomal location of *SrbZIP* gene in *S. rebaudiana*. Table S5: The collinear analysis between *S. rebaudiana* and *A. thaliana*. Table S6: *Cis*-acting elements for *SrbZIP* promoters. Table S7: Homologous genes of the *S. rebaudiana* bZIP gene family in *A. thaliana*. Table S8: Heatmap of *SrbZIP* gene in six *S. rebaudiana* cultivars (110, 11-14, B1188, 023, GP, GX). Table S9: Protein–protein interaction network between SrbZIP proteins and reported bZIP protein involved in terpenoid synthesis. Table S10: Statistical analysis of gene expression after various treatments.
