# Supplementary material for: Genome-Wide Identification and Expression Analysis of bZIP Family Genes in Stevia rebaudiana
Source: Genes (Basel). 2023 Oct 9;14(10):1918. doi: 10.3390/genes14101918 (PMC10606749; doi:10.3390/genes14101918)
Supplement: Supplementary file 1 [file genes-14-01918-s001.zip › Supplementary Materials/Supplementtary Information.pdf]

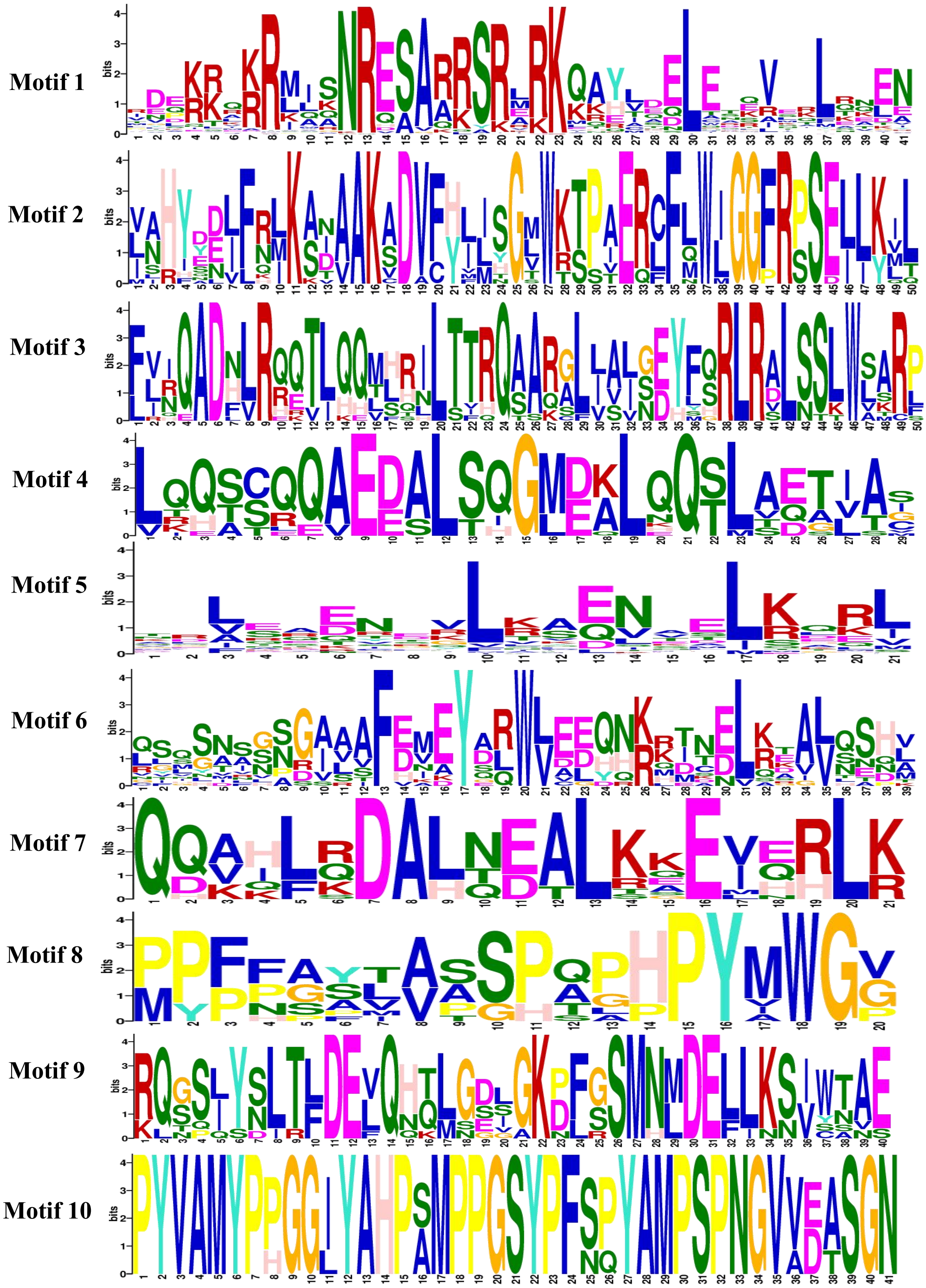

Figure S1. Protein motifs in the bZIP members. The colorful boxes delineate different motifs.

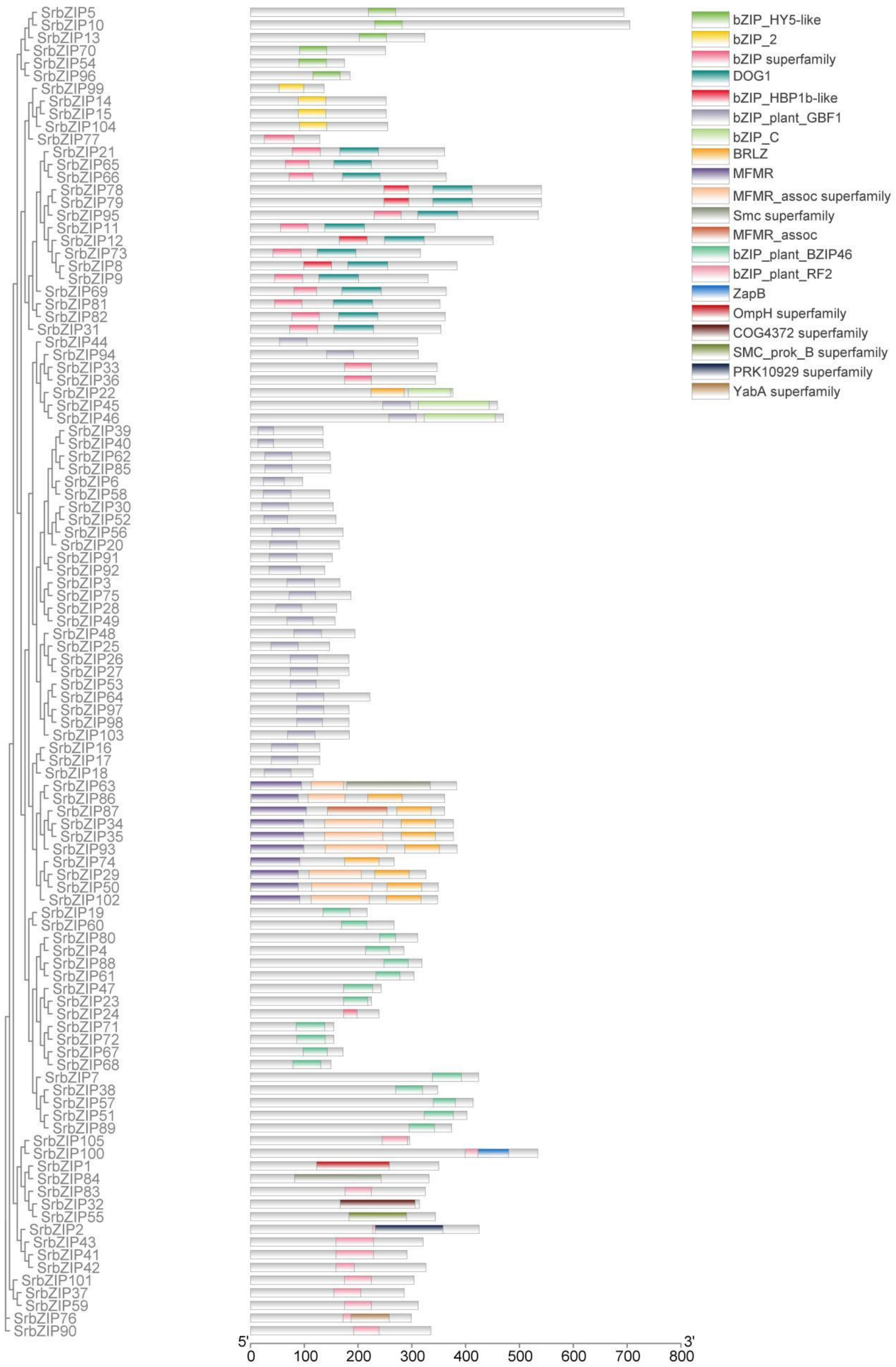

Figure S2. Phylogenetic tree and protein domain. The colorful boxes delineate different domains.

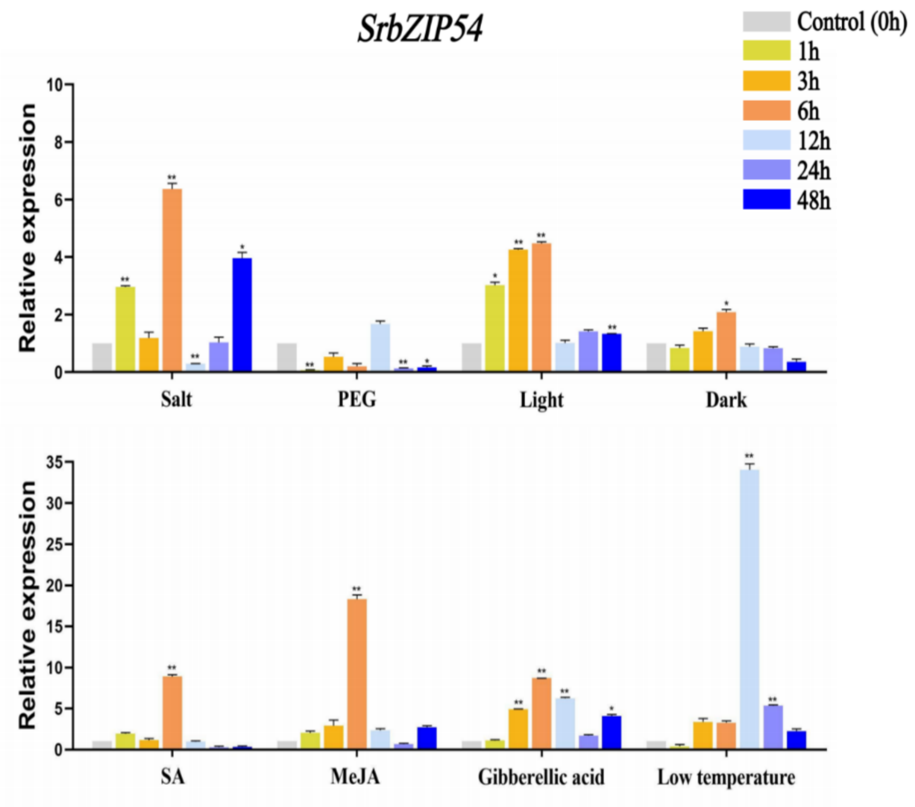

Figure S3. Expression patterns of *SrbZIP54* gene in response to salt, PEG, light, dark, SA, MeJA, gibberellic acid and low temperature treatment. The expression level at 0 h was set as 1. Error bars indicate SE of three biological and technical replicates, and significant differences between treatment group and control group are denoted by asterisk(s) ($^{**}p < 0.01$, $^{*}p < 0.05$)

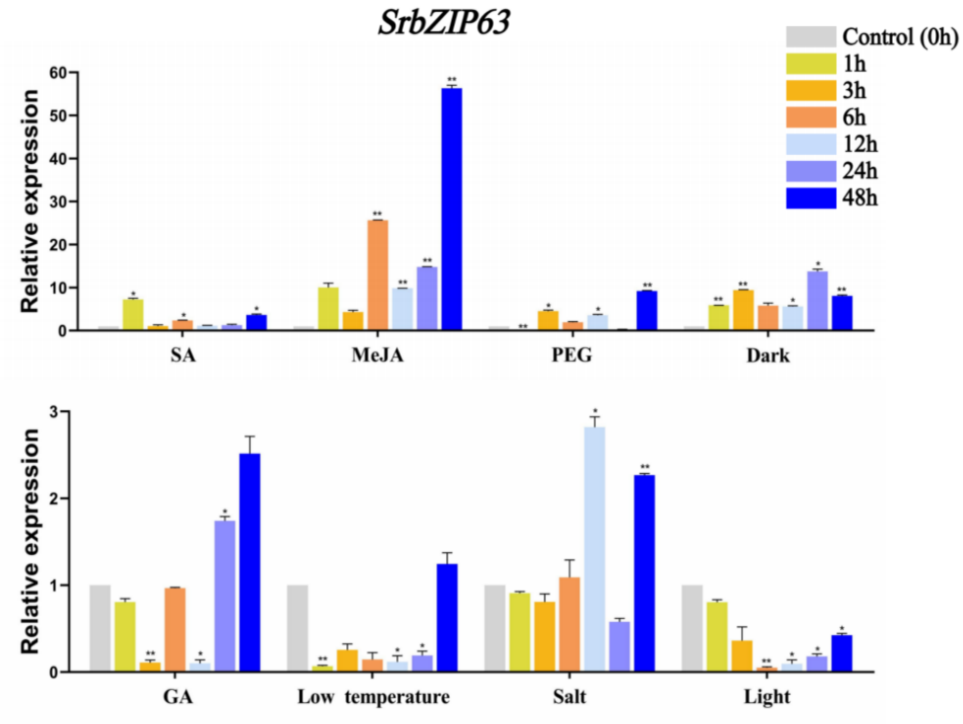

Figure S4. Expression patterns of *SrbZIP63* gene in response to salt, PEG, light, dark, SA, MeJA, gibberellic acid and low temperature treatment. The expression level at 0 h was set as 1. Error bars indicate SE of three biological and technical replicates, and significant differences between treatment group and control group are denoted by asterisk(s) (** $p < 0.01$, * $p < 0.05$)

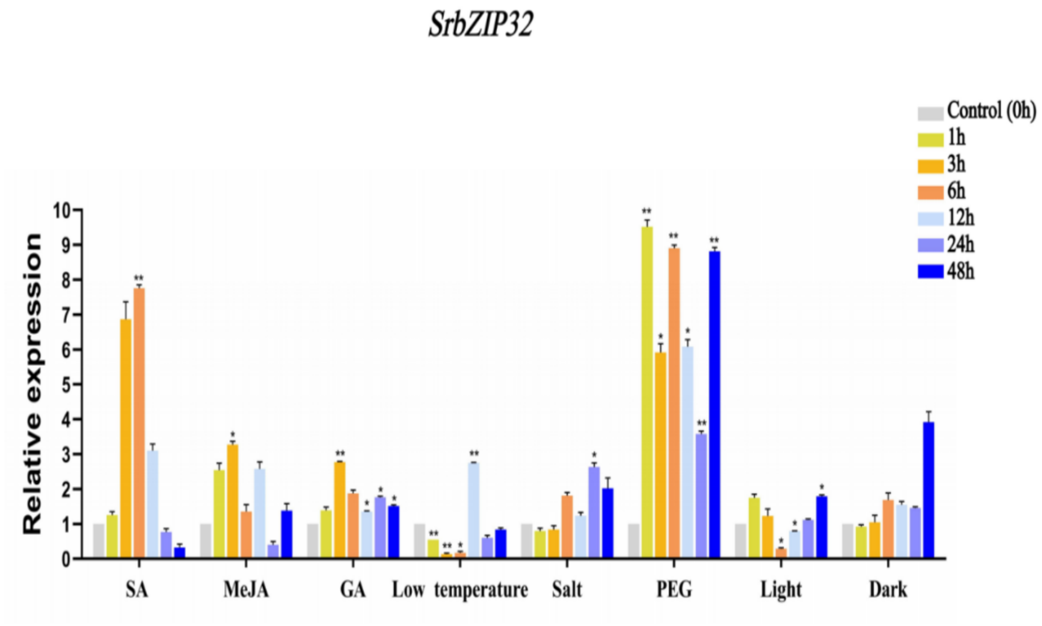

Figure S5. Expression patterns of *SrbZIP32* gene in response to salt, PEG, light, dark, SA, MeJA, gibberellic acid and low temperature treatment. The expression level at 0 h was set as 1. Error bars indicate SE of three biological and technical replicates, and significant differences between treatment group and control group are denoted by asterisk(s) (** $p < 0.01$, * $p < 0.05$)
